# Information flow drives localized morphological differences across neuronal and glial cell types

**DOI:** 10.3389/fncom.2026.1771227

**Published:** 2026-03-11

**Authors:** Paheli Desai-Chowdhry, Alexander B. Brummer, Samhita Mallavarapu, Masai Oakes, Van M. Savage

**Affiliations:** 1 Department of Computational Medicine, University of California, Los Angeles, Los Angeles, CA, United States; 2 Department of Mathematics, Trinity Washington University, Washington, DC, United States; 3 Department of Physics and Astronomy, College of Charleston, Charleston, SC, United States; 4 Department of Computer Science, Tufts University, Medford, MA, United States; 5 Department of Ecology and Evolutionary Biology, University of California, Los Angeles, Los Angeles, CA, United States; 6 Santa Fe Institute, Santa Fe, NM, United States

**Keywords:** axons, biological scaling theory, cell-type classification, dendrite, glia, machine learning, neuron branching, neuron morphology

## Abstract

Neuron processes—axons and dendrites—have distinct branching patterns related to their biological function in the brain and body. Other non-neuronal cells in the nervous system, glia, also have characteristic branching morphologies. Our previous work has used biological scaling theory to connect branching patterns in neurons to biophysical function such as energy or conduction time minimization and material constrants in a compact, unifying mathematical model. Here, we use functionally relevant structural parameters related to asymmetric branching patterns extracted from our model as features in machine-learning classification methods to highlight differences between different types of neurons and glia as well as between healthy and diseased cells. Notably, we find that parameters related to information flow vary with position in the cell—that is, relative proximity of each branching junction to the soma (cell body) or synapses. We find that for some neuronal and glial cell type comparisons, such as comparisons between medium spiny neuron (MSN) dendrites, incorporating relative branching junction location significantly improves the performance of machine-learning classification methods. Our results imply that differences in information flow across cells drive specific morphological changes that correspond to localized regions of neuronal and glial cells. The promise of our methods and results lay foundation for future studies classifying neuronal and glial cells based on pathology, using our asymmetric scale factors and relative branching junction location as potential biomarkers to identify particular diseases based on both structural differences and the underlying differences in function.

## 1 Introduction

Neurons are the fundamental structural units of the nervous system, connecting to one another through their branching processes—axons and dendrites—that allow them to transmit information in the form of electrical and chemical signals. There is a vast diversity of different types of cells that have different morphological forms and biological functions in the nervous system circuitry (Johnston and Wu, 1995). Arguably, the first attempt at cell-type classification in neuroscience is credited to the neuroanatomist Santiago Ramón

y Cajal, who made detailed drawings of the morphological forms of a range of neuronal cell types across species and attempted to comparatively analyze them, arriving at a set of biophysical functional principles that dictate neuron morphology (Ramón y Cajal, 1909). While Ramón y Cajal's work focused on qualitative distinctions across these cell types, more recent work has made use of increasingly quantitative technology to analyze distinctions and to establish a quantitative structure-function correspondence (Cuntz et al., 2010; Chklovskii and Stevens, 2002; Desai-Chowdhry et al., 2022; Gertler et al., 2008; Lu and Yang, 2017; Akram et al., 2022). A major goal in cell-type classification in neuroscience is to establish a correspondence between the different criteria that distinguish cell types from one another such as morphological, physiological, connective/topological, and molecular properties.

Another major goal is to better understand how disease affects these properties in different cell types, and whether there are disease-specific alterations that are related to particular cell types (Zeng and Sanes, 2017). In order to understand the structure, function, and pathology of neuronal cells, it is also important to understand the context in which these cells exist. About half of brain cells are comprised of non-neuronal nervous system cells, called glia. Glial cells are a broad class of cells consisting of the subcategories microglia, astrocytes, and oligodendrocytes (Gellner et al., 2016). For a long time, it was thought that they were simply glue for neurons without any specific function of their own (Jäkel and Dimou, 2017), although Ramón y Cajal hypothesized about their function and realized that more advanced experimental techniques would be required to test his conjectures (García-Marín et al., 2007). More recent research has revealed a range of functions that make them integral to brain function and even information processing. Oligodendrocytes are essential in providing myelin sheaths that protect neuron processes and increase the conduction velocity of information transfer (Peters, 2002). Astrocytes are a key part of synapses—the connections between neurons—that play important roles in regulating synaptic transmission and plasticity (Araque and Navarrete, 2010). They also play a role in the signaling that controls blood flow and metabolism in the brain (Attwell et al., 2010). Microglia are key in generating immune responses and maintaining homeostasis in the central nervous system. They are very sensitive to the environment and they undergo drastic morphological changes in response to neuronal activity and the presence of pathogens (Szepesi et al., 2018). Glial cells, unlike axons, cannot generate action potentials. However, they are electrically active and they communicate with neurons, and a significant amount of energy consumption in the brain contributes to maintaining their resting potentials (Attwell and Laughlin, 2001). Microglia and astrocytes in particular have been shown to respond to electrical stimulation (Gellner et al., 2016). Although their functions are vastly different, they have branching processes that are comparable to neurons, and allow for a similar method of quantitative morphological analysis.

Brummer et al. (2021) analyzed the structure-function correspondence between other types of branching biological networks—such as blood vessels, lungs, and plants—by combining machine-learning classification techniques with a biologically informed mathematical theory that relates vessel branching structure to functional properties that govern resource transport and supply. Rather than simply classifying cells based on arbitrary structural quantities, the connection of these features to function based on the theory provides insights into the structure-function correspondence (Brummer et al., 2021). Further work uses parameters extracted from this theoretical model as features in classification methods that separate cancerous tissue from healthy vessel tissue, providing evidence for the promise of these parameters as potential imaging biomarkers to identify tumors (Brummer and Savage, 2021). Although recent studies have used machine-learning methods to classify between different types of neuronal and glial cell types (Akram et al., 2022), the features they use are purely structural, with no explicit relation to function, and mechanistic insight into how these features relate to differences in function is missing. Moreover, while these studies focus on length as morphometric features classifying cell types (Akram et al., 2022), the caliber of neuronal and glial processes is an important structural feature in that it relates to information flow. Since we have previously built a theory relating neuron morphology to function inspired by this mathematical framework (Desai-Chowdhry et al., 2022, 2023), a promising approach to address cell-type classification as well as potential disease-related alterations in neuronal and glial cells is to combine this theory with machine-learning methods to perform a comparative analysis across cell types. Here, we conduct such an analysis for dendrites and glial cells and propose future studies using our parameters to classify between healthy and diseased cells.

## 2 Theory

Our model considers the tradeoffs among biological functions that neuron structures are evolved to optimize. One important evolutionary function of neuronal networks is the transfer of large amounts of information between brain regions in a short amount of time (Laughlin and Sejnowski, 2003). At the individual cell level, the varied morphological forms observed for neurons are various adaptations to basic principles such as limiting signal time delay (Ramón y Cajal, 1909). Thus, it is important to consider conduction time as a key evolutionary principle that governs neuronal branching structures. We formulate this model based on the dependence of conduction velocity on fiber radius and myelination, using principles set forth by Hodgkin (1954) and Rushton (1951). In addition to optimizing solely conduction time velocity in neurons, there are additional costs due to signaling in the brain that consumes a substantial amount of energy (Attwell and Laughlin, 2001), suggesting that energy expenditure is another important factor that constrains neuron structure. Previous work has shown that the relationship between metabolic rate and conduction time plays an important role in determining axon function in species across scales of body size (Wang et al., 2008). This leads to the West, Brown, Enquist (WBE) framework, which relies on the assumption that resource distribution networks are optimized such that the energy used to transport resources is approximately minimized (West et al., 1997). Our model includes both conduction time and energy efficiency while also incorporating additional factors such as material costs and space-filling (Ramón y Cajal, 1909). Synthesizing these ideas leads to a unifying model that can predict various morphological structural parameters for axons and dendrites across a range of cell types (Desai-Chowdhry et al., 2022, 2023).

**Figure 1:**
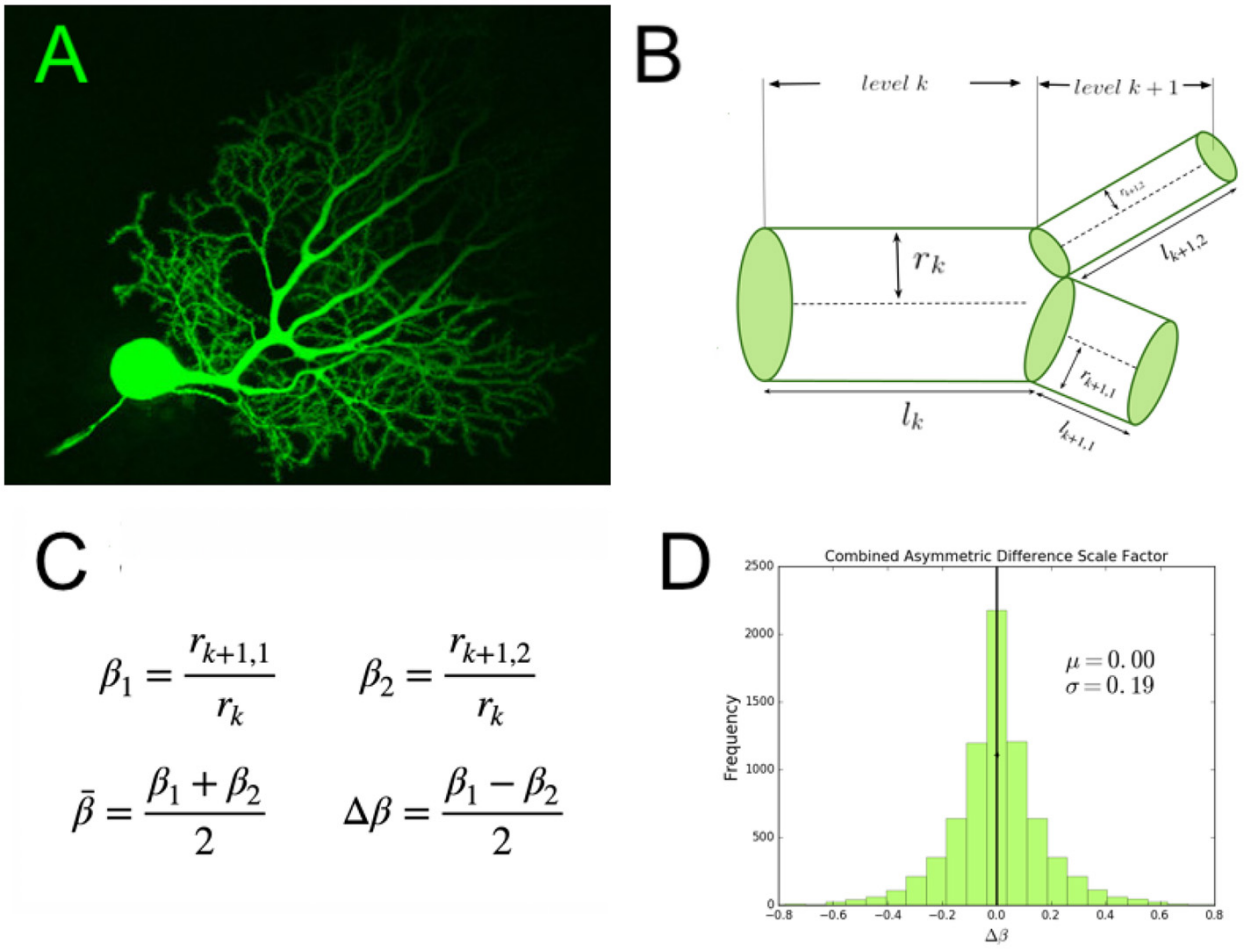
**(A)** An image of a mouse cerebellar Purkinje neuron and its dendritic branching structure. This image was obtained using confocal microscopy and Lucifer yellow fluorescent dye. We have cropped this image that is available on CellImageLibrary.Org, distributed by Maryann Martone, Diana Price, and Andrea Thor (Martone et al., 2002). **(B)** A diagram of a branching junction as part of a hierarchical branching network with successive branching levels, illustrating asymmetric branching junctions. **(C)** Definitions of asymmetric scale factors, *β*_1_ and *β*_2_, and average and difference scale factors, $\bar{\beta }$ and Δ*β*, **(D)** A quantification of the branching asymmetry present across cell types, as measured by the difference scale factor, Δ*β*, where the most symmetric values lie at a value of 0.

We represent neurons as hierarchically branching information processing networks, with successive branching levels that decrease in radius and length according to a scaling relationship. We define *β* as the scaling relationship between the daughter and parent widths, $\frac{r_{k+1}}{r_{k}}$. Figure 1 illustrates this with a representative image and a diagram of a branching junction. Since these branching junctions are often asymmetric—that is, the two daughter branches are not equal to one another—there are two separate scaling ratios that correspond to each of the daughter branches, *β*_1_ and *β*_2_. Based on these two quantities, we can define the average scale factor as $\bar{\beta }=\frac{\beta_{1}+\beta_{2}}{2}$ and the difference scale factor as $\text{Δ}\beta =\frac{\beta_{1}-\beta_{2}}{2}$ (shown in Figure 1C) based on conventions in previous work (Brummer et al., 2017). If we define *β*_1_ as the scaling ratio corresponding to the larger branch, we can describe *β*_1_ and *β*_2_ in terms of the average and absolute value difference scale factors as in Equation 1.


$$\begin{array}{l} \beta_{1}=\bar{\beta }+|\text{Δ}\beta |;\beta_{2}=\bar{\beta }-|\text{Δ}\beta | \end{array}$$ (1)

Thus, we can think of |Δ*β*| as a measure of the magnitude of the asymmetry, or the amount of shift away from the average. Figure 1D shows a distribution of Δ*β* in data combined across a range of cell types and species, preserving the sign as well as the magnitude to show variance around the symmetric case in both directions.

We predict how biological function such as information processing and space-filling govern the branching structure of neurons by optimizing a mathematical cost function subject to a set of constraints, which allows us to obtain theoretical predictions for structural parameters that are the best possible given the biological constraints of the physical system (Boyd and Vandenberghe, 2004). Here, we choose a cost function that minimizes conduction time delay and energy consumption (represented by power loss) that is subject to computational, biological, and physical constraints. The biophysical constraints are represented as functions and added to the expressions to be minimized, allowing us to use the method of undetermined Lagrange multipliers to optimize this overall objective function (Riley et al., 2006).


$$\begin{array}{l} C=\alpha P+\left(1-\alpha \right)T+\underset{i}{\sum }\lambda_{i}f_{i}\left(r_{k},l_{k},k,N,n,\epsilon \right) \end{array}$$ (2)

In this general function *C*, the parameter α can be varied between 0 and 1 to consider the tradeoff between the two principles, *P*, power loss, and *T*, conduction time delay, and the *f*_*i*_ functions are constraint functions, representing biological quantities such as material costs that are held constant during the optimization. We can define *P* as the power lost due to dissipation, which relates to the decay of signals traveling in dendrites from the synapses to the cell body. For a neuronal network, we define the power loss by the equation, $P=I_{0}^{2}R_{net}$, where *I*_0_ is the ionic current and *R*_*net*_ is the resistance to current flow in the network. Because we are focusing on average, large scale quantities across the full extent of the neuron and need to consider a coarse-grained average of signal propagation, we can reasonably approximate axons and dendrites as wires through which current flows and encounters resistance from the neuron fiber. The resistance is given by $R_{k}=\frac{\rho l_{k}}{A_{k}}$, where *A*_*k*_ is the cross sectional area of the wire, and *l*_*k*_ is the length of the segment at that level. The parameter ρ is the intrinsic resistivity of the axon or dendrite, and we assume that ρ is constant, meaning that the material is uniform (Johnston and Wu, 1995). Approximating axons and dendrites as cylinders, the cross-sectional area is $\pi r_{k}^{2}$ for level k, and the resistance is $R_{k}=\frac{\rho l_{k}}{\pi r_{k}^{2}}$. Following standard practice, we have absorbed all physical constants into the Lagrange constants, and the magnitude of these terms do not affect the theoretical predictions.

For an asymmetric branching junction, we define the power loss across the branching network based on recursion using the scaling ratios *β*_1_ and *β*_2_ as well as the analogous length scaling relationships, defined as $\gamma =\frac{l_{k+1}}{l_{k}}$. Using our expressions for these quantities based on the average and difference scale factors in Equation 1, we can describe *P* as


$$\begin{array}{l} P=R_{N,TOT}\overset{N}{\underset{k=0}{\sum }}\left(\overset{N-1}{\underset{j=k}{\prod }}\left[\frac{{\left(\bar{\beta_{j}}+|\text{Δ}\beta_{j}|\right)}^{2}}{\bar{\gamma_{j}}+|\text{Δ}\gamma_{j}|}+\frac{{\left(\bar{\beta_{j}}-|\text{Δ}\beta_{j}|\right)}^{2}}{\bar{\gamma_{j}}+|\text{Δ}\gamma_{j}|}\right]\right) \end{array}$$ (3)

In our analysis, we fix the length scale factor, $\text{Δ}\gamma =\frac{\gamma_{1}-\gamma_{2}}{2}$, to always be positive. This enforces the following sign convention on the difference scale factor for radius. Consequently, when Δ*β* > 0, one child branch will be both wider and longer than the other child branch. When Δ*β* < 0, one child branch will be wider and shorter than the other child branch. These two scenarios correspond to *positive* and *negative* asymmetric branching and provide a visual way to interpret our results. Here, we focus on branch width rather than length, meaning our results are meaningful in terms of the magnitude but not the direction of asymmetry. For the length scaling to be correctly interpreted, we need to use an alternative (Newberry et al., 2015; Brummer et al., 2021; Bentley et al., 2013; Desai-Chowdhry et al., 2022) labeling scheme for branching networks, such as Horton-Strahler labeling. We expand upon this in the Discussion.

Here, we focus on dendrites and glia, which are associated with passive cable attenuation as a key principle rather than action potentials (Chklovskii and Stevens, 2002; Rall, 1977; Gellner et al., 2016). This is associated with the principle *P*, which is related to a voltage drop. We confirm this by looking at the range of scaling ratios and exponents in the data and noticing that they all fall within the range of predictions for specific cases of the function *C* that focus on minimizing power loss, *P*, as predicted in our previous work (Desai-Chowdhry et al., 2022, 2023). Our function *T*, time delay, as described in our model is associated more with active conduction, or action potentials. We associate this principle more with axons, as they are designed to transmit large amounts of energy in a short amount of time (Rall, 1977). Since we do not analyze axons as extensively in this study, we omit the expression for *T*. More information on this principle can be found in our previous work (Desai-Chowdhry et al., 2023). We elaborate on this in the Discussion.

**Figure 2:**
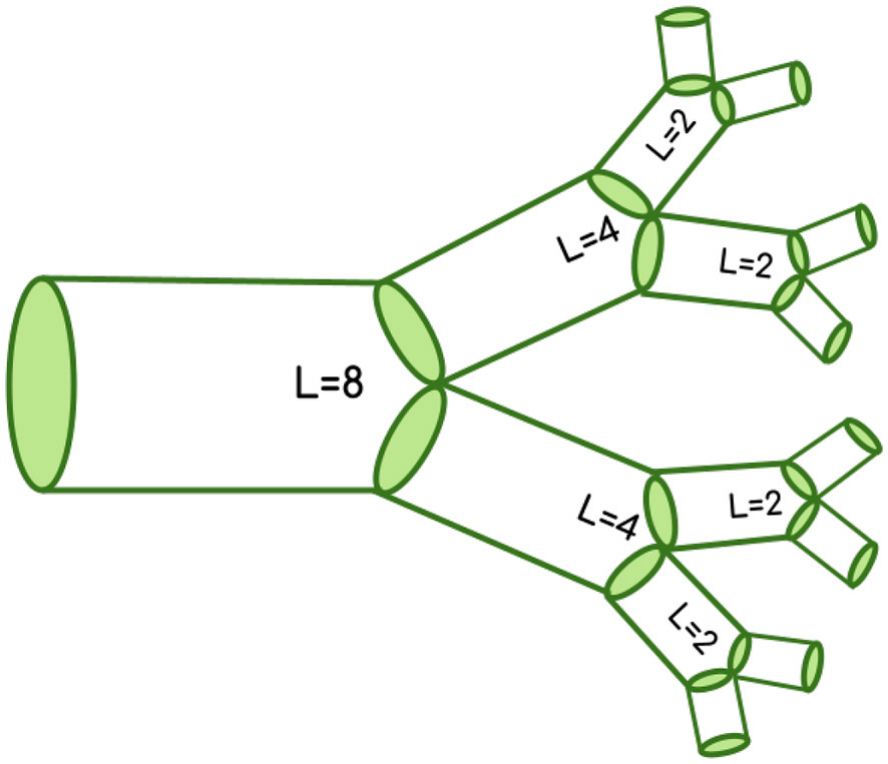
Leaf number visualization. The leaf number, *L*_*n*_, is defined as the number of tips that are distal to each branch. For example, the branches near the tips have a leaf number of 2, and this number increases as we move further from the tips. We incorporate the relative leaf number as a feature to improve classification among cell types. The relative leaf number normalizes the data using the maximum absolute leaf number (*L* = 8 in this image), meaning that *L*_*n,rel*_ values of 0 are closest to the soma, or furthest from the tips.

It is clear that the parameters $\bar{\beta }$ and Δ*β* as defined by this model contribute to information flows through these networks. Although previous work on other types of biological networks focused on these parameters as features to classify between networks (Brummer et al., 2021), here, we explore the incorporation of another feature into our classification. In order to quantify the distance of a branching junction relative to the soma and the synapses, we can use an established measure called leaf number that has been used to study scaling in dendritic branching (Liao et al., 2021). We will refer to the leaf number as *L*_*n*_ throughout this paper and will later use a relative measure, *L*_*n,rel*_, to normalize this parameter across cells to allow for comparisons based on distance from the soma. The leaf number is defined as the number of tips that are distal to each branch. The leaf number at the tips will be equal to 0, and the leaf number will be greatest near the soma. Figure 2 illustrates leaf numbering. For each pair of radius scaling ratios in the data, we have a corresponding leaf number of the parent branch of the junction. Our previous work has shown that the most asymmetric branching junctions occur closest to the tips or the synapses. Moreover, there are different functional principles governing the structure at different regions of the cells (Desai-Chowdhry et al., 2023). Here, we argue that leaf number provides us with essential information about these structures and their correspondence with function. For some comparisons, incorporating leaf number plays a greater role in distinguishing the two groups, as the classification methods perform better with the inclusion of *L*_*n,rel*_ as a feature. Our results thus suggest that the morphological distinctions between cells are driven by information flow at localized cell regions.

## 3 Methods

The morphological data we analyze in this study is taken from NeuroMorpho.Org (Ascoli et al., 2007), an online database with a large amount of morphological data reconstructed from neuron and glia images for a range of cell types and species. In this study, we focus on four different types of dendrites—motoneurons, purkinje cells, medium spiny neurons (MSNs), and pyramidal cells—and two different types of glial cells—astrocytes and microglia—along with some axon data. The quantitative morphological data is extracted from images by tracing neuron image stacks using computational methods, some manual and some automatic, that were obtained using a range of microscopic and staining techniques for *in vitro* neurons sliced at regular intervals. We analyze a total of 160 individual images and 6689 branching points. The motoneurons were from cats (Cullheim et al., 1987), mice (Branchereau et al., 2016), and rats (Rotterman et al., 2014). The Purkinje cells were from mice (Chen et al., 2013; Ljungberg and Watt, 2017). The medium spiny neurons were from mice (Goodliffe et al., 2018). The pyramidal neurons were from humans (Koch and Jones, 2016; Berg et al., 2020). The astrocytes were from mice (Litvinchuk et al., 2018; Clavreul et al., 2019; Ligneul et al., 2019) and rats (Canchi et al., 2017). The microglia were from mice (Ativie et al., 2018; Rossetti et al., 2018; Abdolhoseini et al., 2019) and rats (Althammer et al., 2020; Megjhani et al., 2015). The axons were from mice (Badea et al., 2012).

The data is stored in text files by pixel, where each pixel contains a pixel ID number, *x*, *y*, and *z* spatial coordinates, the radius (based on the distance from the location of the pixel to the edge of the process on each side), and a parent pixel ID number referring to the previous pixel to which each pixel connects. For this analysis, we convert this pixel-based data to branch-based data, defining the branch points in the pixel data by identifying the pairs of pixels where the parent pixel ID numbers are separated by 2 or more. Thus, we obtain branch-based data with a list of branch ID numbers and corresponding parent branch ID numbers. For each branch, we take the average radius of each of the pixels belonging to that branch to assign a radius value.

For each branch, we can find the scaling ratio, *β*, by dividing its radius by the radius of the parent branch to which it is assigned. We can identify two daughter branches of the same branching junction by identifying branches that share the same parent branch. From the two daughters, we can extract the parameters $\bar{\beta }$ and Δ*β* by computing $\bar{\beta }=\frac{\beta_{1}+\beta_{2}}{2}$ and $\text{Δ}\beta =\frac{\beta_{1}-\beta_{2}}{2}$. We filter this data by removing all pairs of daughter for which any one of the daughters has *β* ≥ 0.999. The values that are very close to 1 are likely an artifact due to the resolution limit of the images; after a certain level, all of the radius value are equal to the pixel size of the image, leading to the computation of *β*≈1. Filtering these data removes large peaks and symmetries observed in the raw data that are due to these limitations of the measurements.

In this study, we are also interested in looking at the position of these branching junctions relative to the soma and the synapses. We measure this based on the leaf number, *L*_*n*_—the number of distal branches at every branching junction. We calculate this by looping through the branch data, identifying the tips as the branches that are not the parent branches of other branches, and assigning each of the remaining branches the sum of the number of distal tips of its daughters branches. However, in order to normalize these *L*_*n*_ values to allow for comparisons between different cells and cell-types, we define a new parameter, *L*_*n,rel*_. For each cell, from the list of *L*_*n*_ values, we identify the maximum value, *L*_*n, max*_, defining the branch furthest from the tips or closest to the soma. We define each of the other *L*_*n,rel*_ values at each point i relative to this value, computing $L_{n,rel,i}=log_{2}(\frac{L_{n,max}}{L_{n,i}})$. Thus, for the point at which *L*_*n, max*_ = *L*_*n*_, *L*_*n,rel*_ is equal to 0, defines the soma. For some images, we averaged the values of $\bar{\beta }$ and Δ*β* at each *L*_*n,rel*_ for each image or cell-type in order to aid visualization and discernment of clusters in the data.

For each comparison, we assigned each cell-type a classifier label to be either 0 or 1. The closest set to a balanced set was created by balancing the number of images used for each cell-type, but it should be noted that with this method, it is not possible to control for the number of data points in each image due to the variability of the images. When two cell-types were being compared and more data were available for one type, a subset of data for that type was used for comparison in order to achieve even more balance. Between 70 and 80% of the images were randomly assigned to the training data for each cell type, and the remaining data were assigned to the test set. We used seven different machine-learning classification methods, which we later refer to by abbreviations: Logistic Regression (LR), Support Vector Machine (SVM), K-Nearest Neighbors (KNN), Random Forest (RF), Decision Tree (DT), Naïve Bayes (Bayes), and Neural Networks (NN). These methods were implemented in RStudio. For Logistic Regression, we used the function *glm* to build the model for the classifier. For Support Vector Machine and Naïve Bayes classification, we used the package “e1071,” using the functions *svm* and *naiveBayes*. For SVM, we used the “radial” kernel, which we showed to perform better than other kernels. This is supported by previous results (Akram et al., 2022; Luts et al., 2010). For K-Nearest Neighbors, we used the package “FNN” using the function *knn*, where the number of nearest neighbors, k, was chosen based on local maxima of the performance metrics of classification, accuracy and AUC. For Random Forest classification, we used the package “randomForest,” using the function *randomForest*. For Decision Tree classification, we used the package “rpart,” using the function *rpart*. For Neural Networks, we used the package “neuralnet,” using the functions *neuralnet* and *compute*.

These classification methods use the features, either $\bar{\beta }$ and Δ*β* or $\bar{\beta }$, Δ*β*, and *L*_*n,rel*_ and the assigned classifier label in the training set to predict the classifier labels for each of the points in the test set. These predicted values are given in a list of the length of the number of points in the test data. One way to measure the performance of the classification methods is to look at how accurately the points are classified, comparing the predictions to the classifier labels of each of the points in the test set and reporting the percentage of points that are labeled accurately. Here, in Table 1, we report the accuracy measures of each of the classification methods by image, where the classification was performed on the raw branching junction test data for all the images, and the average predicted label for each image was compared to the true label. However, a more useful measure of the performance of these methods is the area under the curve (AUC) of the Receiver Operating Characteristic (ROC) Curve that considers the tradeoff between true positive rates and false positive rates, which is more useful for datasets like these that are not balanced. Tables 2–8 report the performance of the seven classification methods based on the AUC, where the uncertainty measure is based on a 95% confidence interval. The ROC metrics, such as the AUC and confidence intervals, were computed using the package “pROC.” The plots of the ROC curves were created using the package “ggplot2.” The plots of all other visualizations were created using the Python library matplotlib.

**Table 1:** Image based classification accuracy measures.

**Classification/method**	**LR**	**SVM**	**KNN**	**RF**	**DT**	**Bayes**	**NN**
Motoneurons/purkinje 2D	4/6 images	4/6 images	4/6 images	4/6 images	4/6 images	4/6 images	4/6 images
Motoneurons/purkinje 3D	4/6 images	4/6 images	4/6 images	5/6 images	4/6 images	4/6 images	4/6 images
MSNs/pyramidal 2D	13/21 images	13/21 images	13/21 images	17/21 images	14/21 images	13/21 images	10/21 images
MSNs/pyramidal 3D	13/21 images	13/21 images	14/21 images	17/21 images	16/21 images	13/21 images	10/21 images
Motoneurons/pyramidal 2D	3/6 images	4/6 images	4/6 images	4/6 images	4/6 images	4/6 images	4/6 images
Motoneurons/pyramidal 3D	4/6 images	5/6 images	5/6 images	5/6 images	5/6 images	4/6 images	5/6 images
MSNs/purkinje 2D	4/7 images	5/7 images	5/7 images	7/7 images	5/7 images	4/7 images	5/7 images
MSNs/purkinje 3D	7/7 images	6/7 images	6/7 images	7/7 images	7/7 images	4/7 images	7/7 images
Motoneurons/MSNs 2D	3/7 images	4/7 images	5/7 images	6/7 images	4/7 images	4/7 images	3/7 images
Motoneurons/MSNs 3D	7/7 images	7/7 images	7/7 images	7/7 images	7/7 images	7/7 images	7/7 images
Purkinje/pyramidal 2D	3/6 images	3/6 images	3/6 images	3/6 images	3/6 images	3/6 images	3/6 images
Purkinje/pyramidal 3D	3/6 images	3/6 images	4/6 images	4/6 images	5/6 images	4/6 images	4/6 images
Astrocytes/microglia 2D	7/10 images	7/10 images	7/10 images	7/10 images	7/10 images	7/10 images	6/10 images
Astrocytes/microglia 3D	5/10 images	7/10 images	7/10 images	8/10 images	4/10 images	4/10 images	6/10 images

This table shows the accuracy measures of classification using combined data from all the individual data points to classify each image or whole neuron based on average classification of all branching points in the image for each method. Out of all the images with branching junction data that fit our precision requirements for the pixel sizes, we balanced the sets of images and randomly assigned 70%–80% of the images to training data and the remaining images to test data. The numbers of images here for each classification (comparison between two groups) reflects the number of images in those test sets.

**Table 2:** Motoneurons vs. purkinje cells classification area under ROC curve (AUC) measures.

**Features/method**	**LR**	**SVM**	**KNN**	**RF**	**DT**	**Bayes**	**NN**
$\bar{\beta },\text{Δ}\beta$	0.719 ± 0.058	0.700 ± 0.062	0.735 ± 0.055	0.761 ± 0.052	0.695 ± 0.056	0.723 ± 0.057	0.719 ± 0.058
$\bar{\beta },\text{Δ}\beta ,L_{n,rel}$	0.723 ± 0.056	0.766 ± 0.053	0.691 ± 0.058	0.715 ± 0.056	0.729 ± 0.053	0.724 ± 0.057	0.738 ± 0.057

The area under the curve (AUC) values corresponding to the ROC curves show the strongest performance when they are closest to 1. Here, we can see that the Random Forest (RF) method performs the best for 2-dimensional feature space, and adding the additional feature *L*_*n,rel*_ increases the AUC for most methods.

**Table 3:** Medium spiny neurons vs. pyramidal cells classification area under ROC curve (AUC) measures.

**Features/method**	**LR**	**SVM**	**KNN**	**RF**	**DT**	**Bayes**	**NN**
$\bar{\beta },\text{Δ}\beta$	0.574 ± 0.061	0.677 ± 0.060	0.720 ± 0.051	0.846 ± 0.038	0.802 ± 0.042	0.599 ± 0.058	0.531 ± 0.066
$\bar{\beta },\text{Δ}\beta ,L_{n,rel}$	0.760 ± 0.041	0.830 ± 0.043	0.834 ± 0.034	0.912 ± 0.026	0.856 ± 0.031	0.740 ± 0.043	0.766 ± 0.043

The area under the curve (AUC) values corresponding to the ROC curves show the strongest performance when they are closest to 1. Here, we can see that the Random Forest (RF) method performs the best for both 2-dimensional and 3-dimensional feature spaces, and adding the additional feature *L*_*n,rel*_ significantly increases the AUC for all methods.

**Table 4:** Motoneurons vs. pyramidal cells classification area under ROC curve (AUC) measures.

**Features/method**	**LR**	**SVM**	**KNN**	**RF**	**DT**	**Bayes**	**NN**
$\bar{\beta },\text{Δ}\beta$	0.681 ± 0.041	0.816 ± 0.033	0.792 ± 0.036	0.827 ± 0.032	0.793 ± 0.035	0.776 ± 0.038	0.680 ± 0.041
$\bar{\beta },\text{Δ}\beta ,L_{n,rel}$	0.777 ± 0.039	0.870 ± 0.030	0.814 ± 0.039	0.884 ± 0.029	0.851 ± 0.033	0.803 ± 0.038	0.772 ± 0.040

The area under the curve (AUC) values corresponding to the ROC curves show the strongest performance when they are closest to 1. Here, we can see that the Random Forest (RF) method performs the best for both 2-dimensional and 3-dimensional feature spaces, followed closely here by the Suppport Vector Machine (SVM) method, and adding the additional feature *L*_*n,rel*_ increases the AUC for all methods.

**Table 5:** Medium spiny neurons vs. purkinje cells classification area under ROC curve (AUC) measures.

**Features/method**	**LR**	**SVM**	**KNN**	**RF**	**DT**	**Bayes**	**NN**
$\bar{\beta },\text{Δ}\beta$	0.578 ± 0.075	0.599 ± 0.074	0.616 ± 0.073	0.771 ± 0.059	0.639 ± 0.064	0.624 ± 0.072	0.579 ± 0.075
$\bar{\beta },\text{Δ}\beta ,L_{n,rel}$	0.776 ± 0.060	0.884 ± 0.045	0.861 ± 0.048	0.907 ± 0.038	0.854 ± 0.049	0.742 ± 0.064	0.792 ± 0.062

The area under the curve (AUC) values corresponding to the ROC curves show the strongest performance when they are closest to 1. Here, we can see that the Random Forest (RF) method performs the best for both 2-dimensional and 3-dimensional feature spaces, and adding the additional feature *L*_*n,rel*_ significantly increases the AUC for all methods.

**Table 6:** Motoneurons vs. medium spiny neurons classification area under ROC curve (AUC) measures.

**Features/method**	**LR**	**SVM**	**KNN**	**RF**	**DT**	**Bayes**	**NN**
$\bar{\beta },\text{Δ}\beta$	0.586 ± 0.071	0.749 ± 0.056	0.746 ± 0.056	0.805 ± 0.048	0.732 ± 0.054	0.747 ± 0.054	0.549 ± 0.059
$\bar{\beta },\text{Δ}\beta ,L_{n,rel}$	0.885 ± 0.035	0.924 ± 0.029	0.871 ± 0.036	0.952 ± 0.021	0.923 ± 0.027	0.835 ± 0.047	0.886 ± 0.035

The area under the curve (AUC) values corresponding to the ROC curves show the strongest performance when they are closest to 1. Here, we can see that the Random Forest (RF) method performs the best for both 2-dimensional and 3-dimensional feature spaces, and adding the additional feature *L*_*n,rel*_ significantly increases the AUC for all methods.

## 4 Results

**Figure 3:**
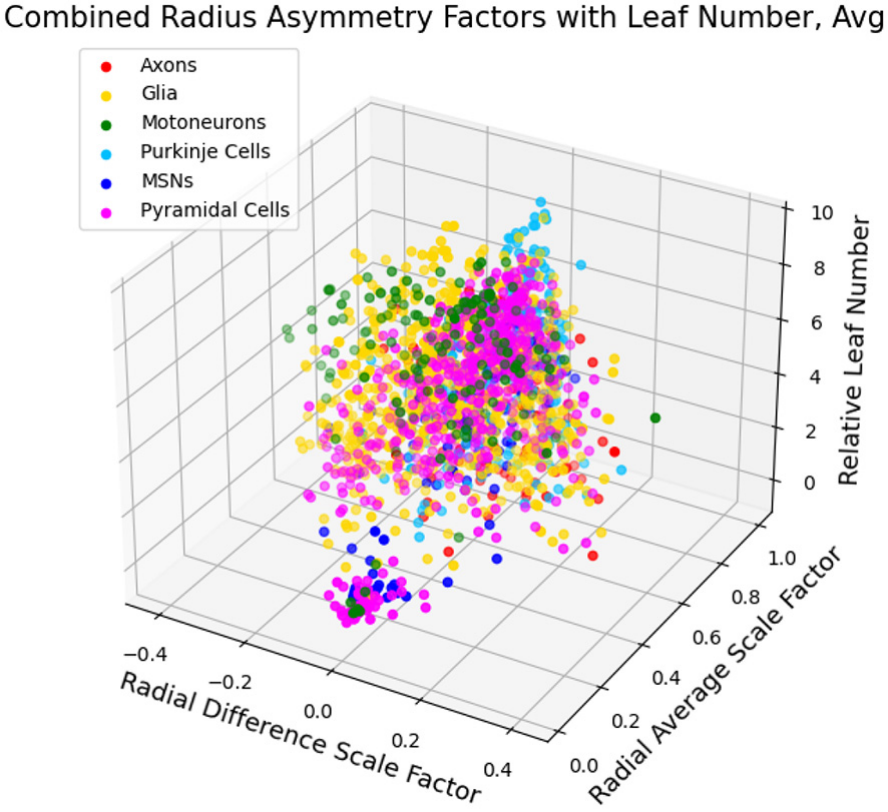
Plots of different cell/process types in the feature space of $\bar{\beta }$, Δ*β*, and *L*_*n,rel*_. For each image, the average $\bar{\beta }$ and Δ*β* are taken at each level, *L*_*n,rel*_. Here, we see clear clusters indicating the distinctions between the different types in this feature space. This overview provides initial support for our subsequent more detailed analysis of the distictions between cell-types in this feature space.

**Figure 4:**
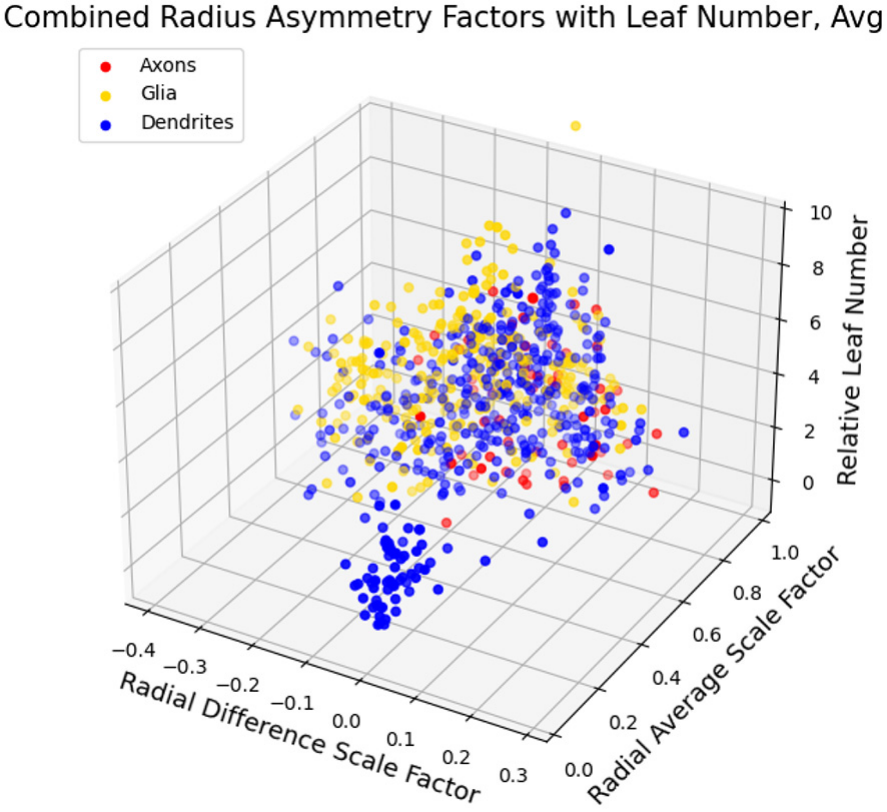
Plots of different cell/process types, clustered by axons, dendrites, and glial cells, in the feature space of $\bar{\beta }$, Δ*β*, and *L*_*n,rel*_. Here, for each cell type, the average $\bar{\beta }$ and Δ*β* are taken at each level, *L*_*n,rel*_. The small cluster localized at low $\bar{\beta }$ and *L*_*n,rel*_ values is unique to dendrites, providing a distinct characteristic property of this process type.

**Figure 5:**
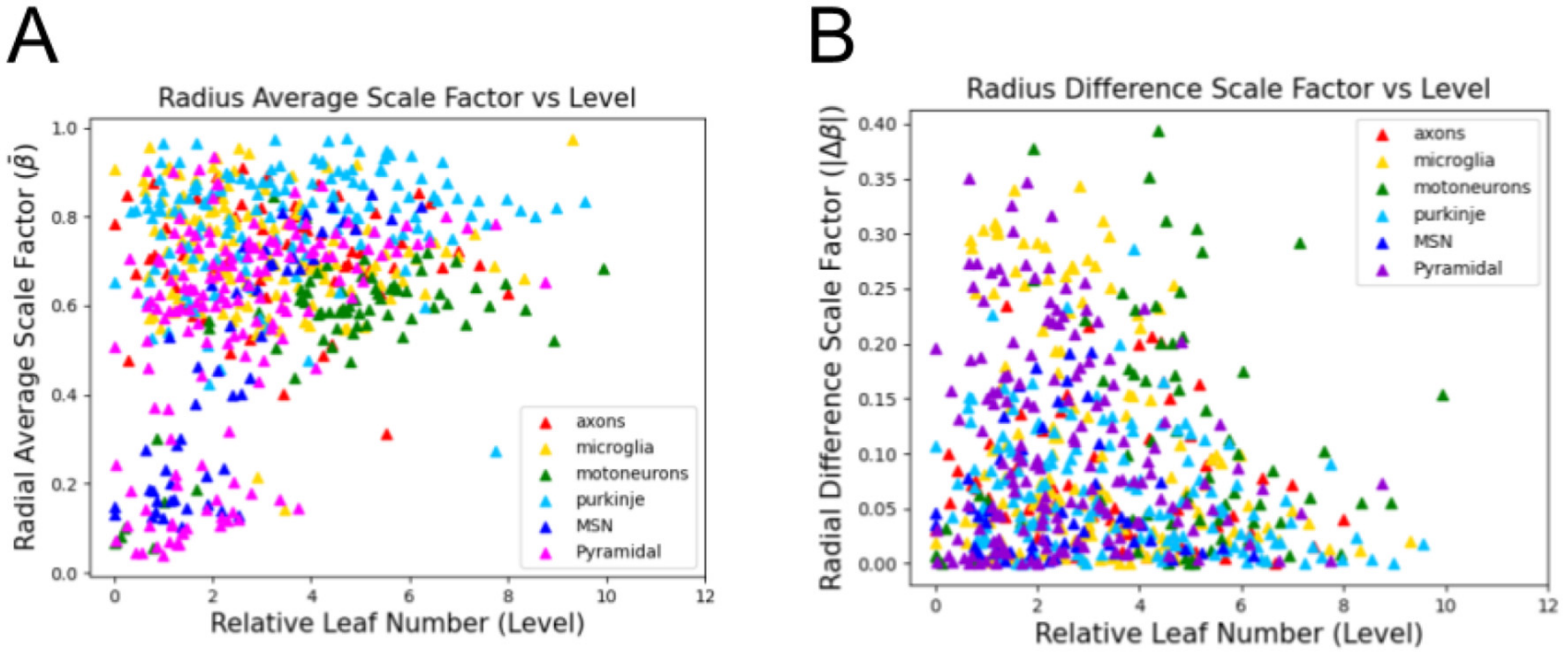
Plots showing the relationship between **(A)** The average scale factor and the relative leaf number and **(B)** The magnitude of the difference scale factor and the relative leaf number. For all cell and process types, there appears to be a general relationship between both $\bar{\beta }$ and *L*_*n,rel*_ as well as between Δ*β* and *L*_*n,rel*_, though these relationships are different from one another. This relationship between the features provides a potential explanation for the relatively stronger performance of Random Forest and Support Vector Machine classification methods.

In Figure 3, we illustrate the feature space of the combined data from a range of different dendritic cell-types, as well as axons and glial cells, based on $\bar{\beta }$, Δ*β*, and *L*_*n,rel*_. We can locate clear clusters that indicate the distinctions between the types based on these features. Note that symmetries observed in the plots of the 2-dimensional feature space are due to the way the data is filtered, and not a feature of the data itself. Figure 4 further indicates the distinctions between dendrites, axons, and glial processes. We observe that out of the cell and process types observed here, the small localized cluster at low $\bar{\beta }$ as well as low *L*_*n,rel*_ values is unique to dendrites. It is important to note that for all of these cell and process types, there appears to be a general relationship between both $\bar{\beta }$ and *L*_*n,rel*_ as well as between Δ*β* and *L*_*n,rel*_, as we show in Figure 5.

We further illuminate the differences between different cell-types within these three groups, comparing dendrites and glial cells first, and then applying these methods to look at distinctions between healthy and diseased cells.

### 4.1 Dendrites

**Figure 6:**
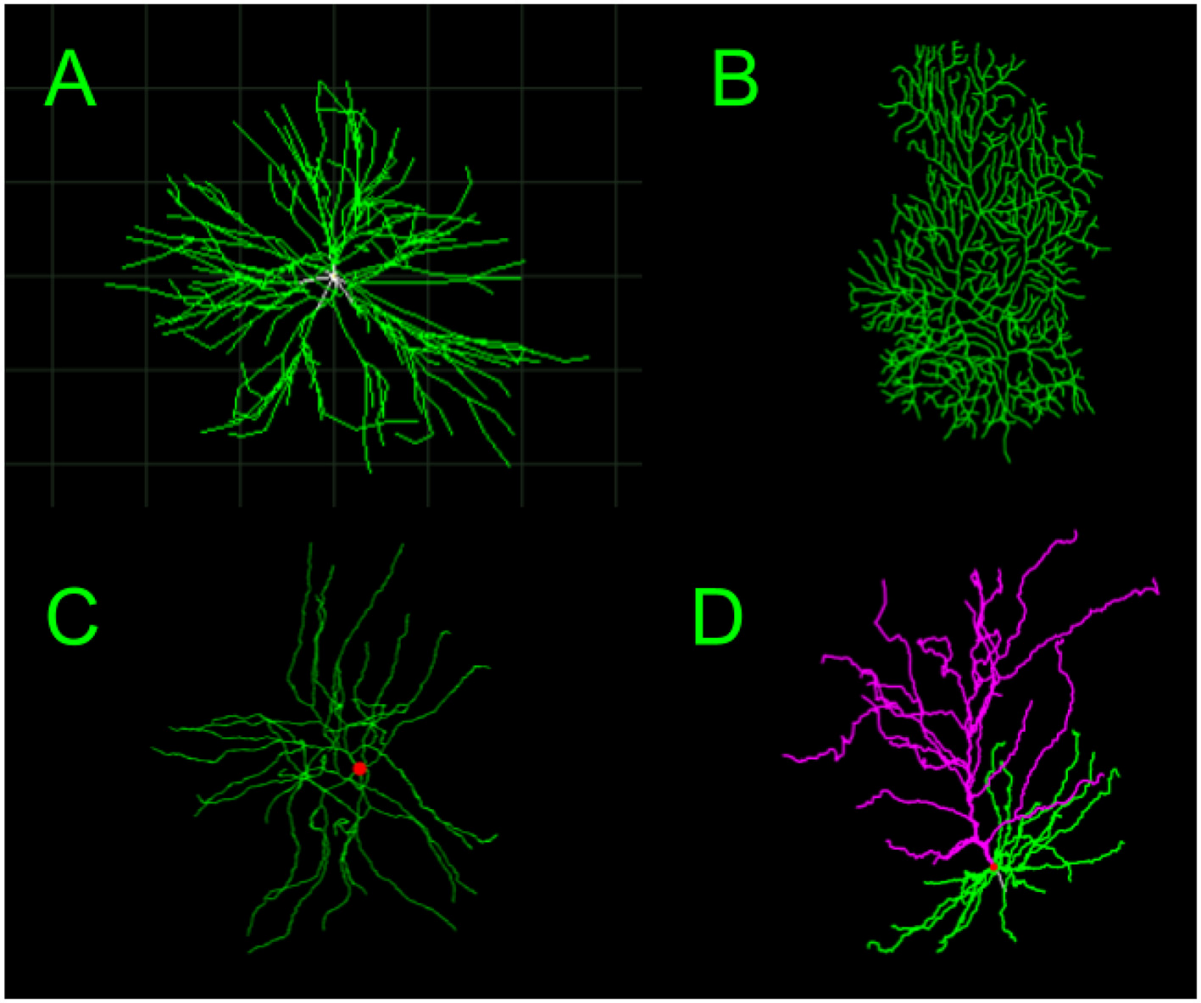
Images showing reconstructions of the images and morphological data from NeuroMorpho.Org (Ascoli et al., 2007) for different dendrite types including **(A)** Motoneurons, from a cat spinal motoneuron (Cullheim et al., 1987). **(B)** Purkinje Cells, from a mouse (Murru et al., 2019), **(C)** Medium Spiny Neurons, from a mouse (Goodliffe et al., 2018), and **(D)** Pyramidal Cells, from a human (Berg et al., 2020).

**Figure 7:**
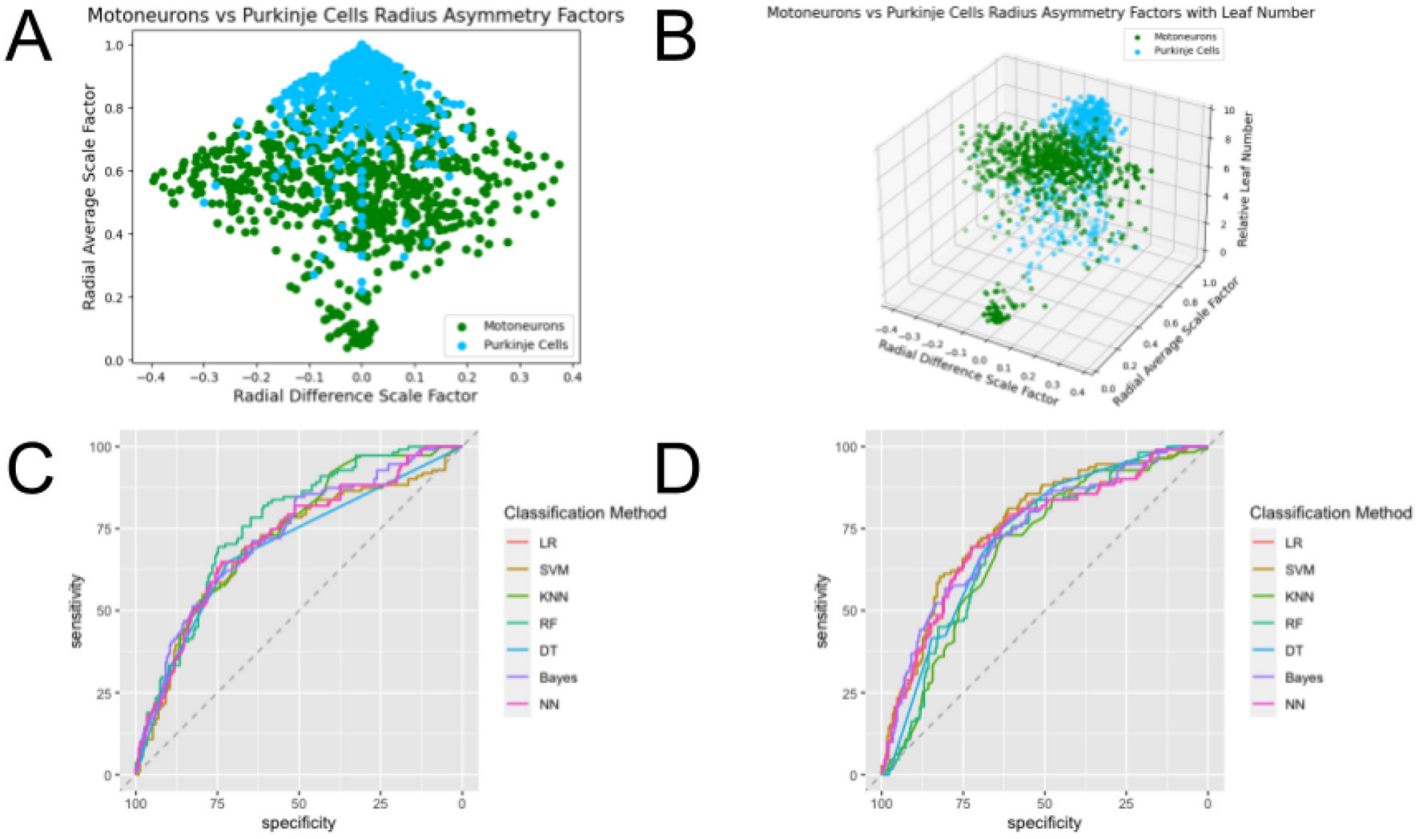
Plots of the feature spaces for comparing two different types of dendrites—Motoneurons and Purkinje Cells—using **(A)** $\bar{\beta }$ and Δ*β* and **(B)** $\bar{\beta }$, Δ*β*, and *L*_*n,rel*_ as features, and plots of the ROC curves illustrating the performance of classification methods using **(C)** $\bar{\beta }$ and Δ*β* and **(D)** $\bar{\beta }$, Δ*β*, and *L*_*n,rel*_ as features. One can observe clear distinctions between the cell types, evidenced by the separate clusters of data points of each of the assigned colors. The clusters observed in the 3-dimensional feature space do not show significantly greater distinctions than those observed for the 2-dimensional feature space. ROC curves show the tradeoff between true positive rates and false positive rates, which is a more accurate assessment of the performance of classifications than accuracy especially for datasets that are not balanced. The area under the curve (AUC) of these ROC curves show the strongest performance when they are closest to 1, and the curves that are closer to the top left corner at the intersection of axes than the horizontal dashed line illustrate better performance. We can observe that the ROC curves for classification in the 3-dimensional feature space do not show significantly better performance than those for classification in the 2-dimensional feature space.

**Figure 8:**
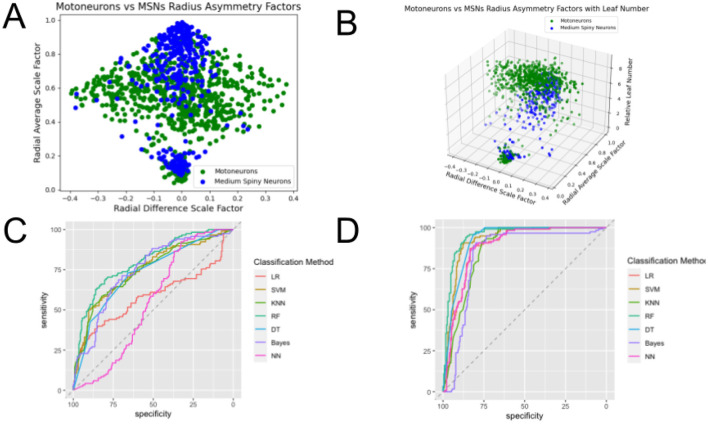
Plots of the feature spaces for comparing two different types of dendrites—Motoneurons and Medium Spiny Neurons—using **(A)** $\bar{\beta }$ and Δ*β* and **(B)** $\bar{\beta }$, Δ*β*, and *L*_*n,rel*_ as features, and plots of the ROC curves illustrating the performance of classification methods using **(C)** $\bar{\beta }$ and Δ*β* and **(D)** $\bar{\beta }$, Δ*β*, and *L*_*n,rel*_ as features. One can observe clear distinctions between the cell types, evidenced by the separate clusters of data points of each of the assigned colors. The clusters observed in the 3-dimensional feature space show greater distinctions than those observed for the 2-dimensional feature space. ROC curves show the tradeoff between true positive rates and false positive rates, which is a more accurate assessment of the performance of classifications than accuracy especially for datasets that are not balanced. The area under the curve (AUC) of these ROC curves show the strongest performance when they are closest to 1, and the curves that are closer to the top left corner at the intersection of axes than the horizontal dashed line illustrate better performance. This comparison shows the greatest classification performance. We can observe the green ROC curves, corresponding to the Random Forest (RF) method, consistently on top of the set of curves, showing its superior performance in classification. Moreover, we can observe that the ROC curves for classification in the 3-dimensional feature space show significantly better performance than those for classification in the 2-dimensional feature space.

**Figure 9:**
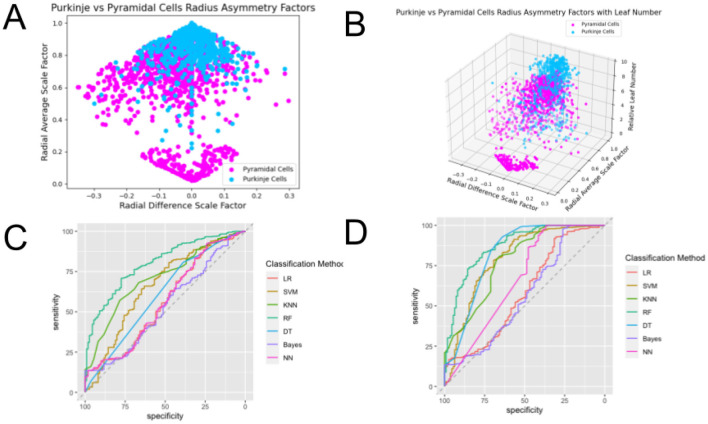
Plots of the feature spaces for comparing two different types of dendrites—Purkinje cells and Pyramidal Cells—using **(A)** $\bar{\beta }$ and Δ*β* and **(B)** $\bar{\beta }$, Δ*β*, and *L*_*n,rel*_ as features, and plots of the ROC curves illustrating the performance of classification methods using **(C)** $\bar{\beta }$ and Δ*β* and **(D)** $\bar{\beta }$, Δ*β*, and *L*_*n,rel*_ as features. One can observe clear distinctions between the cell types, evidenced by the separate clusters of data points of each of the assigned colors. The clusters observed in the 3-dimensional feature space show significantly greater distinctions than those observed for the 2-dimensional feature space. ROC curves show the tradeoff between true positive rates and false positive rates, which is a more accurate assessment of the performance of classifications than accuracy especially for datasets that are not balanced. The area under the curve (AUC) of these ROC curves show the strongest performance when they are closest to 1, and the curves that are closer to the top left corner at the intersection of axes than the horizontal dashed line illustrate better performance. This comparison shows the weakest classification performance out of the dendrite comparisonns. We can observe the green ROC curves, corresponding to the Random Forest (RF) method, consistently on top of the set of curves, showing its superior performance in classification. Moreover, we can observe that the ROC curves for classification in the 3-dimensional feature space show better performance than those for classification in the 2-dimensional feature space, particularly for Support Vector Machine (SVM).

Here, we compare four different types of dendrites—Motoneurons, Purkinje cells, Medium Spiny Neurons, and Pyramidal cells—for a total of six comparisons. Visual representations of these cells, reconstructed from the image morphological data on NeuroMorpho.Org (Ascoli et al., 2007) are shown in Figure 6. Tables 2–7 show the performance of seven different classification methods. We first compare these dendrites using $\bar{\beta }$ and Δ*β* as features and using $\bar{\beta }$, Δ*β*, and *L*_*n,rel*_ as features. The performance of these methods are measured by the area under curve (AUC) of the ROC curves. For simplicity, we have included three representative comparisons in Figures 7–9. The visual representations of the rest of the six comparisons can be found in the Supplemental text. The data in Tables 2–7 are based on the combined branching point data for all images. Classification of the cell-types in the test sets based on the whole images, or the average prediction of each of the points in the images, is shown in Table 1.

**Table 7:** Purkinje vs. pyramidal cells classification area under ROC curve (AUC) measures.

**Features/method**	**LR**	**SVM**	**KNN**	**RF**	**DT**	**Bayes**	**NN**
$\bar{\beta },\text{Δ}\beta$	0.563 ± 0.061	0.660 ± 0.060	0.701 ± 0.049	0.802 ± 0.042	0.607 ± 0.051	0.532 ± 0.060	0.564 ± 0.062
$\bar{\beta },\text{Δ}\beta ,L_{n,rel}$	0.585 ± 0.063	0.794 ± 0.053	0.784 ± 0.051	0.873 ± 0.038	0.824 ± 0.052	0.566 ± 0.064	0.665 ± 0.060

The area under the curve (AUC) values corresponding to the ROC curves show the strongest performance when they are closest to 1. Here, we can see that the Random Forest (RF) method performs the best for both 2-dimensional and 3-dimensional feature spaces, and adding the additional feature *L*_*n,rel*_ increases the AUC for all methods.

For all of these comparisons, the seven classification methods perform relatively well, and incorporating *L*_*n,rel*_ as an additional feature in the classification improves the performance, though the improvement is most significant for the comparison of Medium Spiny Neurons and all the other three cells. The classification method that consistently performed the best for all comparisons was Random Forest.

### 4.2 Glia

**Figure 10:**
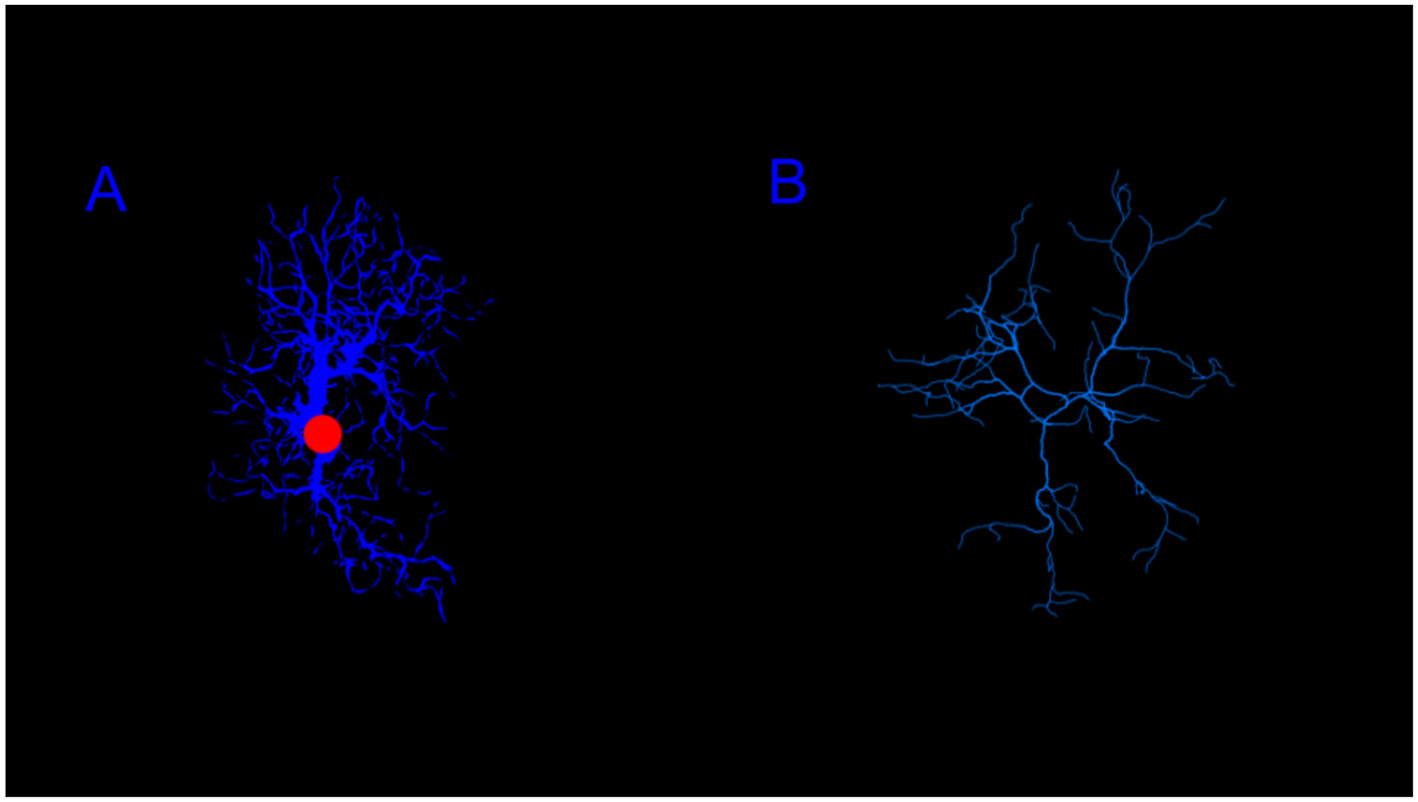
Images showing reconstructions of the images and morphological data from NeuroMorpho.Org (Ascoli et al., 2007) for different glial cell types including **(A)** Astrocytes, from a mouse (Clavreul et al., 2019) **(B)** Microglia, from a mouse (Althammer et al., 2020).

**Figure 11:**
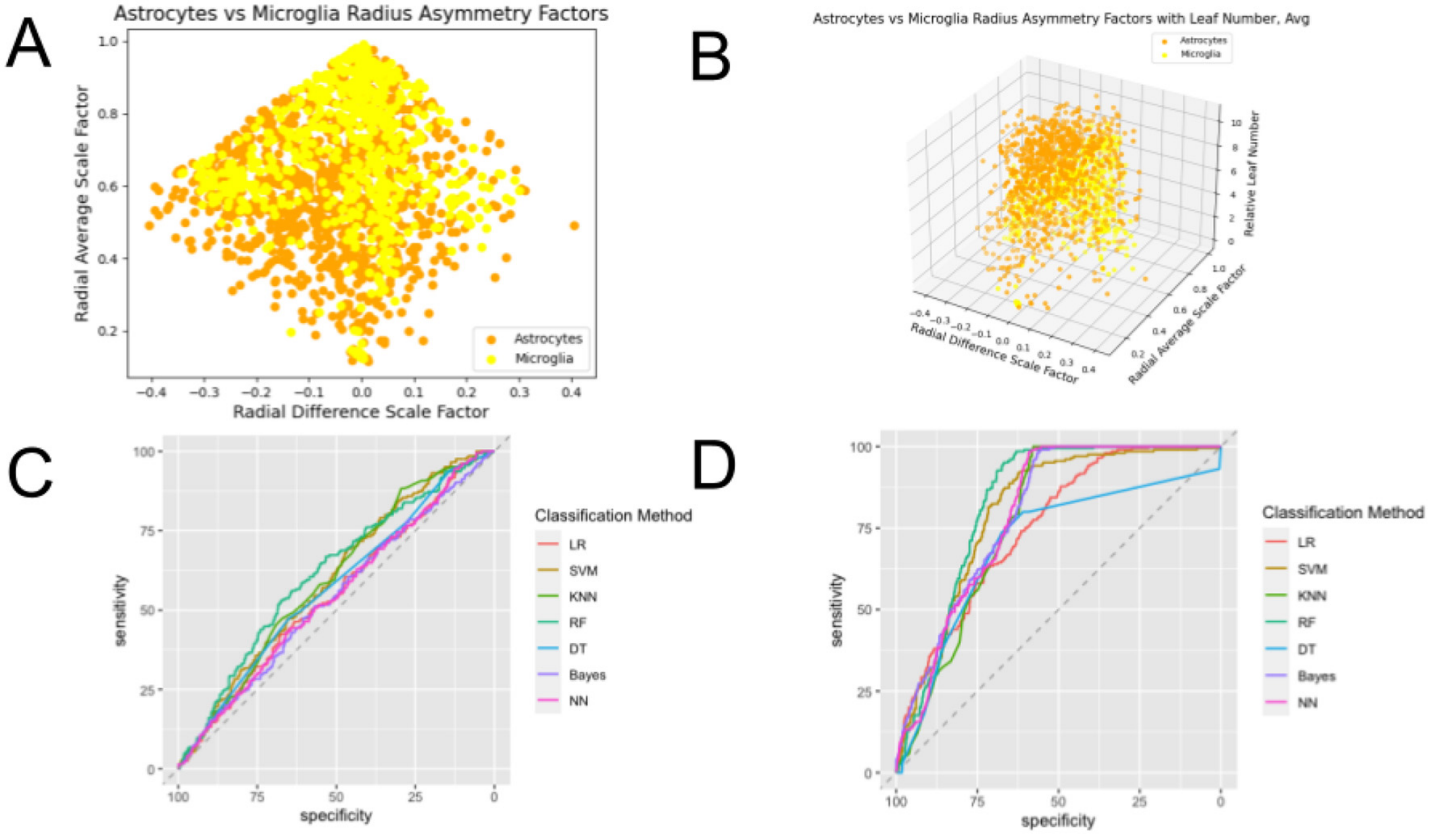
Plots of the feature spaces for comparing two different types of glial cells—astrocytes and microglia—using **(A)** $\bar{\beta }$ and Δ*β* and **(B)** $\bar{\beta }$, Δ*β*, and *L*_*n,rel*_ as features, and plots of the ROC curves illustrating the performance of classification methods using **(C)** $\bar{\beta }$ and Δ*β* and **(D)** $\bar{\beta }$, Δ*β*, and *L*_*n,rel*_ as features. One can observe clear distinctions between the glial cell types, evidenced by the separate clusters of data points of each of the assigned colors. The clusters observed in the 3-dimensional feature space show significantly greater distinctions than those observed for the 2-dimensional feature space. ROC curves show the tradeoff between true positive rates and false positive rates, which is a more accurate assessment of the performance of classifications than accuracy especially for datasets that are not balanced. The area under the curve (AUC) of these ROC curves show the strongest performance when they are closest to 1, and the curves that are closer to the top left corner at the intersection of axes than the horizontal dashed line illustrate better performance. We can observe the green ROC curves, corresponding to the Random Forest (RF) method, consistently on top of the set of curves, showing its superior performance in classification. Moreover, we can observe that the ROC curves for classification in the 3-dimensional feature space show significantly better performance than those for classification in the 2-dimensional feature space.

Here, we compare two different types of glial cells—astrocytes and microglia. Visual representations of these cells, reconstructed from the image morphological data on NeuroMorpho.Org (Ascoli et al., 2007) are shown in Figure 10. Table 8 shows the performance of seven different classification methods. We compare these glial cells using both the two-dimensional and three-dimensional feature spaces as previously defined. The performance of these methods are measured by the area under curve (AUC) of the ROC curves. These visual representation of the results are shown in Figure 11. The data in Table 8 are based on the combined branching point data for all images. Classification of the cell-types in the test sets based on the whole images, or the average prediction of each of the points in the images, is shown in Table 1.

**Table 8:** Astrocytes vs. microglia classification area under ROC curve (AUC) measures.

**Features/method**	**LR**	**SVM**	**KNN**	**RF**	**DT**	**Bayes**	**NN**
$\bar{\beta },\text{Δ}\beta$	0.549 ± 0.045	0.596 ± 0.043	0.596 ± 0.043	0.615 ± 0.044	0.571 ± 0.044	0.538 ± 0.046	0.544 ± 0.045
$\bar{\beta },\text{Δ}\beta ,L_{n,rel}$	0.753 ± 0.035	0.804 ± 0.031	0.775 ± 0.030	0.827 ± 0.027	0.702 ± 0.042	0.776 ± 0.031	0.794 ± 0.030

The area under the curve (AUC) values corresponding to the ROC curves show the strongest performance when they are closest to 1. Here, we can see that the Random Forest (RF) method performs the best for both 2-dimensional and 3-dimensional feature spaces, and adding the additional feature *L*_*n,rel*_ significantly increases the AUC for all methods.

Here, incorporating *L*_*n,rel*_ as an additional feature in the classification significantly improves the performance of all seven classification methods, as the data are nearly indistinguishable for the two-dimensional feature space, but high AUC and accuracy values for most methods using the three-dimensional space. The classification method that performed the best was Random Forest.

## 5 Discussion

Overall, radius scaling ratios, $\bar{\beta }$ and Δ*β*, perform well in classifying between the 4 different types of dendrites, suggesting that information flow is a driving force in distinguishing different types of cells. For dendrites, signals generally travel from the synapses—the junctions between two cells—back to the cell body, and relate to the information flow of signals through the network through passive cable attentuation (Rall, 1977). For most of these comparisons, these parameters related to information flow are generally sufficient to classify between them, as the improvement in the performance of all classification methods by including the third feature (relative leaf number) is minimal. In particular, the distinctions between Motoneurons and Purkinje Cells (shown in Figure 7) and between Motoneurons and Pyramidal Cells show minimal improvements. The cells with higher $\bar{\beta }$ values show on average less change in the radius from daughter to parent branches. It makes sense, for example, that these values are higher on average for Purkinje cells as compared to motoneurons because Purkinje cells have more extensive branching trees and signals must travel through many branching junctions in order to reach the soma. Less changes in the width means that the conduction velocity remains more steady throughout the trajectory of a signal through the network. The observation that on average, the Δ*β* values remain closer to 0 for Purkinje cells is consistent with the fact that these structure tend to be symmetric.

For all the comparisons involving Medium Spiny Neurons, the incorporation of *L*_*n,rel*_ as a feature more significantly improved performance across classification methods. A representative example is shown in Figure 8. This suggests that for Medium Spiny Neurons, there might be more region-specific differences in the information flow through dendrites. For classification between Purkinje Cells and Pyramidal Cells (shown in Figure 9), both methods perform poorly for both 2-dimensional and 3-dimensional feature spaces, but some methods, such as Support Vector Machine, Decision Tree, and Neural Networks, perform better for the 3-dimensional feature space, and some methods, such as K-Nearest Neighbors and Random Forest, perform well for both spaces.

Interestingly, for glial cells (shown in Figure 11), the distinction between the two cell-types—astrocytes and microglia—is not significant for the 2-dimensional feature space, but the addition of *L*_*n,rel*_ as a feature very significantly boosts the performance of all classification methods. This suggests that for glial cells, there might be more region-specific differences in the information flow through their processes.

Due to the promise of these methods in classifying different types of neuronal and glial cell types, we apply these methods to look at distinctions between healthy cells and diseased cells in order to attempt to extract insights about the pathology. Our preliminary results are available in the Supplementary material. Further studies are necessary to validate these results.

Out of all of the seven machine learning classification methods utilized in this study, Random Forest performed consistently better than all other methods, both in terms of accuracy of classifying images and in terms of the tradeoff between true positive rates and false positive rates of classifying individual data points. The high performance of the Random Forest method over other standard methods has been previously noted (Couronné et al., 2018). The next method that tended to perform better than others was Support Vector Machine, using a radial kernel, which has been shown to perform well on biological data (Akram et al., 2022; Luts et al., 2010). An important advantage of both these methods is that they perform well when there is a relationship between the features (Osisanwo et al., 2017; Sarica et al., 2017). As we observe in Figure 5, there appears to be a nonlinear relationship between both $\bar{\beta }$ and *L*_*n,rel*_ as well as between Δ*β* and *L*_*n,rel*_. This might explain the higher performance of RF and SVM over the Naïve Bayes method, for example, because Naïve Bayes makes the key assumption that the features are independent of one another (Osisanwo et al., 2017; Sen et al., 2020). Moreover, methods such as K-Nearest Neighbors are sensitive to noise, which might explain their variable performance (Osisanwo et al., 2017). Random Forest methods are known to be more accurate compared to Decision Tree methods, as they are a combination of multiple decision trees (Couronné et al., 2018).

In this analysis, we have chosen to focus on radius scaling ratios. Although length scaling ratios might provide additional insights into the distinctions between these cells in relation to other biological properties, such as the ways in which these branching processes fill space, the branch length measurements are not accurately characterized, as also previously reported for vascular scaling (Newberry et al., 2015) as well as other types of plant and animal networks (Brummer et al., 2021; Bentley et al., 2013). Recent work suggests Horton-Strahler labeling—where the first level begins at the tips, and higher levels are determined when two branches of the same level combine—may yield better estimates of branch length scaling, as it has been previously applied to neurons and other biological networks (Brummer and Savage, 2021; Turcotte et al., 1998; Vormberg et al., 2017). In future work, we plan to investigate how this alternative labeling scheme for branch lengths improves the characterization of length scaling ratios, and whether further insights into the distinctions between these cells can be gleaned from studying asymmetries in the lengths of daughter branches.

While our results are promising and suggest that this method of analysis is useful for extracting new insights about neuronal and glial cells, we are limited in the amount of morphological data available at a high enough precision to allow for this method of analysis. In particular, our ability to analyze axons was severely limited by the limited number of images available with the level of precision required for this analysis, as axons are generally thinner than dendrites and their widths are often smaller than the pixel size or resolution limit of the images. Recent studies have made progress in axon reconstruction methods (Li et al., 2025) might lead to a greater amount of usable data for this purpose in the future. Consequently, our study makes a strong case for collecting more high-precision morphological data across neuronal and glial cell types, applying these methods to larger datasets, using more modern classification methods, and reproducing these results as well as extending them further. In future work, we aim to employ new classification methods that have been developed and shown to outperform traditional methods for neuron morphology classification (He et al., 2023; Jiang et al., 2025) using our functionally-informed features for classification. Moreover, previous work has hypothesized that due to similarities in the diameters of dendrites and actin-rich filopodia, dendrite branching and formation could be driven by cytoskeletal elements (Liao et al., 2021), which we have not considered in our work. As this work is developed further in experimental groups, we aim to incorporate these elements into future workings of our model to increase its biological accuracy.

In conclusion, our study combines machine-learning methods with a functionally informed structural model of neuronal and glial processes to not only classify between different types of cells, but to understand the functional basis behind those differences in structure. Although machine-learning is a tool that often obscures mechanistic insight into how models are able to make predictions, our features—$\bar{\beta }$ and Δ*β*—have specific connections to functional principles related to information flow (Brummer et al., 2021).

We introduce *L*_*n,rel*_ as another feature in our model, providing insight into the localization of functionally driven structural differences across neuronal and glial cell types as well as potential disease-related alterations. As more and more data are becoming available using high precision microscopy, such as new electron microscopy datasets by the FlyEM project at Janelia (Shinomaya et al., 2019; Schefferetal, 2020), and artificial intelligence to automate the reconstruction process from neuron images (Zhang et al., 2024; Nourollah et al., 2024; Liao et al., 2025), many more opportunities to apply these methods to even larger datasets at higher resolutions and across more cell types will arise. Moreover, we aim to extend this work to look at distinctions between controls and animal models of mental health disorders from morphological data that has recently become available (Bartmann, 2022). Recent work has applied machine learning methods to identify structure-function correspondences at both macroscopic and microscopic levels of the brain, identifying characteristic features of Autism Spectrum Disorder (ASD) in both network-level *in vivo* structural MRI data and detailed axon data (Yazdanbakhsh et al., 2025; Lokray et al., 2025), and we aim to contribute to these results through analogous studies of dendritic branching structures using our features as potential biomarkers. In this paper, we have scratched the surface of attempting to understand the function of glial cells, about which current knowledge is limited, as well as disease-related morphological changes and functional underpinnings of those changes. Our results illustrate the promise of these methods to further tackle these important problems that will help us better understand the basic building blocks of the nervous system.

## Data Availability

The original contributions presented in the study are included in the article/Supplementary material, further inquiries can be directed to the corresponding author.
