# Supplementary material for: Information flow drives localized morphological differences across neuronal and glial cell types
Source: Front Comput Neurosci. 2026 Mar 11;20:1771227. doi: 10.3389/fncom.2026.1771227 (PMC13013023; doi:10.3389/fncom.2026.1771227)
Supplement: Supplementary file 1 [file Data_Sheet_1.pdf]

Data sources from NeuroMorpho.Org

Table 1. Data Sources for Axons - Machine Learning Classification

Cell Type	Region	Species	Archive Name	File Name
sensory	Peripheral Nervous System	mouse	Badea	Badea2012Fig6A-C-L
sensory	Peripheral Nervous System	mouse	Badea	Badea2012Fig6A-C-R
sensory	Peripheral Nervous System	mouse	Badea	Badea2012Fig6B
sensory	Peripheral Nervous System	mouse	Badea	Badea2012Fig6E-I-L
sensory	Peripheral Nervous System	mouse	Badea	Badea2012Fig6E-I-M
sensory	Peripheral Nervous System	mouse	Badea	Badea2012Fig6E-I-R
sensory	Peripheral Nervous System	mouse	Badea	Badea2012Fig6G
sensory	Peripheral Nervous System	mouse	Badea	Badea2012Fig6H
sensory	Peripheral Nervous System	mouse	Badea	Badea2012Fig6J
sensory	Peripheral Nervous System	mouse	Badea	Badea2012Fig6L
sensory	Peripheral Nervous System	mouse	Badea	Badea2012Fig7C

Table 2. Data Sources for Glia - Astrocytes - Machine Learning Classification

Cell Type	Region	Species	Archive Name	File Name
astrocyte	hippocampus	rat	King	A1-CA1-L-C63x1zACR2
astrocyte	hippocampus	rat	King	A1-CA1-L-F63x1zACR1
astrocyte	hippocampus	rat	King	A2-CA1-L-A63x1zACR5
astrocyte	hippocampus	rat	King	A2-CA1-L-D63x1zACR1
astrocyte	hippocampus	rat	King	A2-CA1-R-H63x1zACR2
astrocyte	hippocampus	rat	King	A3-CA1-L-A63x1zACR3_1
astrocyte	hippocampus	rat	King	B4-CA1-L-A63x1zCell4ACR
astrocyte	hippocampus	rat	King	B4-CA1-L-C63x1zCell1ACR
astrocyte	hippocampus	rat	King	C6-CA1-L-D3x1zACR4
astrocyte	hippocampus	mouse	Zheng	PS19-C3aR-KO-astrocytes1
astrocyte	hippocampus	mouse	Zheng	PS19-C3aR-KO-astrocytes2
astrocyte	hippocampus	mouse	Zheng	PS19-astrocytes
astrocyte	neocortex	mouse	Hernandez-Garzon	S11_2_1
astrocyte	neocortex	mouse	Hernandez-Garzon	S2_3_1_2
astrocyte	neocortex	mouse	Hernandez-Garzon	S5_1_2
astrocyte	neocortex	mouse	Hernandez-Garzon	S7_1_2
astrocyte	neocortex	mouse	Hernandez-Garzon	S8_2_2
astrocyte	neocortex	mouse	Hernandez-Garzon	S9_2_2
astrocyte	basal ganglia	mouse	Hernandez-Garzon	Series027_1ok
astrocyte	basal ganglia	mouse	Hernandez-Garzon	Series029_3ok

Table 3. Data Sources for Glia - Microglia - Machine Learning Classification

Cell Type	Region	Species	Archive Name	File Name
microglia	hippocampus	mouse	Bilkei-Gorzo	2_48
microglia	hippocampus	mouse	Bilkei-Gorzo	2_50
microglia	hippocampus	mouse	Bilkei-Gorzo	3_40
microglia	hippocampus	mouse	Bilkei-Gorzo	6_41
microglia	spinal cord	mouse	Morara	A11aCSF_Iba1-Z_32-26
microglia	spinal cord	mouse	Morara	A20CGRP_Iba1-Z_103-11
microglia	hypothalamus	rat	Althammer	PVN10_microglia_10
microglia	hypothalamus	rat	Althammer	PVN10_microglia_15
microglia	hypothalamus	rat	Althammer	PVN10_microglia_19
microglia	hypothalamus	rat	Althammer	PVN10_microglia_27
microglia	hypothalamus	rat	Althammer	PVN11_microglia_12
microglia	hypothalamus	rat	Althammer	PVN9_microglia_26
microglia	hypothalamus	rat	Althammer	PVN9_microglia_8
microglia	Central Nervous System	mouse	Abdolhoseini_Kluge	cell001_GroundTruth
microglia	Central Nervous System	mouse	Abdolhoseini_Kluge	cell004_GroundTruth
microglia	Central Nervous System	mouse	Abdolhoseini_Kluge	cell006_GroundTruth
microglia	Central Nervous System	mouse	Abdolhoseini_Kluge	cell010_GroundTruth
microglia	neocortex	rat	Roysam	farsight1083
microglia	neocortex	rat	Roysam	farsight1102
microglia	hippocampus	mouse	Bilkei-Gorzo	tgLPSF1684Iba1o_7

Table 4. Data Sources for Motoneurons - Machine Learning Classification

Cell Type	Region	Species	Archive Name	File Name
motoneurons	spinal cord	rat	Alvarez	Alvarez-Control-Cell-3
motoneurons	spinal cord	rat	Alvarez	Alvarez-Regen-Cell-4
motoneurons	spinal cord	mouse	Branchereau_Cattaert	BranchereauCattaert_ScienficReport2016_E17_5MN_x60
motoneurons	spinal cord	mouse	Branchereau_Cattaert	MartinNeurobiolDesease2013_E17_5MN_SOD1G93A_x60
motoneurons	spinal cord	mouse	Branchereau_Cattaert	MartinNeurobiolDesease2013_E17_5MN_WildType_x60
motoneurons	spinal neurons	cat	Burke	v_e_moto1
motoneurons	spinal neurons	cat	Burke	v_e_moto2
motoneurons	spinal neurons	cat	Burke	v_e_moto3
motoneurons	spinal neurons	cat	Burke	v_e_moto4
motoneurons	spinal neurons	cat	Burke	v_e_moto5
motoneurons	spinal neurons	cat	Burke	v_e_moto6

Table 5. Data Sources for Purkinje Cells - Machine Learning Classification

Cell Type	Region	Species	Archive Name	File Name
purkinje	cerebellum	mouse	Dusart	Purkinje-slice-ageP35-1
purkinje	cerebellum	mouse	Dusart	Purkinje-slice-ageP35-2
purkinje	cerebellum	mouse	Dusart	Purkinje-slice-ageP35-3
purkinje	cerebellum	mouse	Dusart	Purkinje-slice-ageP35-4
purkinje	cerebellum	mouse	Dusart	Purkinje-slice-ageP37-5
purkinje	cerebellum	mouse	Dusart	Purkinje-slice-ageP43-6
purkinje	cerebellum	mouse	Watt	SCA6_P10_C1
purkinje	cerebellum	mouse	Watt	SCA6_P10_C5
purkinje	cerebellum	mouse	Watt	SCA6_P11_C2
purkinje	cerebellum	mouse	Watt	SCA6_P11_C3
purkinje	cerebellum	mouse	Watt	SCA6_P12_C4

Table 6. Data Sources for D1-type dopamine receptor-expressing Medium Spiny Neuron (MSN) - Machine Learning Classification

Cell Type	Region	Species	Archive Name	File Name
D1 MSN	basal ganglia	mouse	Luebke	Apr12IR2a
D1 MSN	basal ganglia	mouse	Luebke	Apr12IR2a
D1 MSN	basal ganglia	mouse	Luebke	Apr26IR2e
D1 MSN	basal ganglia	mouse	Luebke	Apr29IR2a
D1 MSN	basal ganglia	mouse	Luebke	Apr29IR2b
D1 MSN	basal ganglia	mouse	Luebke	May24IR2b
D1 MSN	basal ganglia	mouse	Luebke	May5IR2a
D1 MSN	basal ganglia	mouse	Luebke	May9IR2a
D1 MSN	basal ganglia	mouse	Luebke	May9IR3b
D1 MSN	basal ganglia	mouse	Luebke	May9IR3d
D1 MSN	basal ganglia	mouse	Luebke	Nov3IR2c
D1 MSN	basal ganglia	mouse	Luebke	Nov8IR3a
D1 MSN	basal ganglia	mouse	Luebke	Nov9IR2a
D1 MSN	basal ganglia	mouse	Luebke	Nov9IR3c
D1 MSN	basal ganglia	mouse	Luebke	WT-D1-Jul19IR3d-stitch
D1 MSN	basal ganglia	mouse	Luebke	WT-D1-Jun19IR2a-whole-cell
D1 MSN	basal ganglia	mouse	Luebke	WT-D1-Jun27IR3b-whole-cell
D1 MSN	basal ganglia	mouse	Luebke	WT-D1-Jun5IR3b-whole-cell

Table 7. Data Sources for D2-type dopamine receptor-expressing Medium Spiny Neuron (MSN) - Machine Learning Classification

Cell Type	Region	Species	Archive Name	File Name
D2 MSN	basal ganglia	mouse	Luebke	Apr12IR1b
D2 MSN	basal ganglia	mouse	Luebke	Apr12IR3a
D2 MSN	basal ganglia	mouse	Luebke	Apr12IR3b
D2 MSN	basal ganglia	mouse	Luebke	Apr19IR3f
D2 MSN	basal ganglia	mouse	Luebke	Apr26IR1a
D2 MSN	basal ganglia	mouse	Luebke	Apr29IR1c
D2 MSN	basal ganglia	mouse	Luebke	Apr29IR3a
D2 MSN	basal ganglia	mouse	Luebke	Nov3IR2a_1
D2 MSN	basal ganglia	mouse	Luebke	Nov3IR2d
D2 MSN	basal ganglia	mouse	Luebke	Nov3IR2e
D2 MSN	basal ganglia	mouse	Luebke	Nov3IR3b
D2 MSN	basal ganglia	mouse	Luebke	Nov8IR2b
D2 MSN	basal ganglia	mouse	Luebke	Nov9IR3b
D2 MSN	basal ganglia	mouse	Luebke	WT-D2-Jun19IR3a-whole-cell
D2 MSN	basal ganglia	mouse	Luebke	WT-D2-Jun27IR3c-whole-cell
D2 MSN	basal ganglia	mouse	Luebke	WT-D2-June19IR2c

Table 8. Data Sources for Pyramidal Control Cells - Machine Learning Classification

Cell Type	Region	Species	Archive Name	File Name
pyramidal	neocortex	human	Allen Cell Types	H16-03-003-01-18-01_556380191_m
pyramidal	neocortex	human	Allen Cell Types	H16-03-008-11-11-02_601947643_m
pyramidal	neocortex	human	Allen Cell Types	H16-03-008-11-11-03_606347920_m
pyramidal	neocortex	human	Allen Cell Types	H16-06-004-01-04-01_538906745_m
pyramidal	neocortex	human	Allen Cell Types	H16-06-004-01-04-05_556380170_m
pyramidal	neocortex	human	Allen Cell Types	H16-06-004-01-13-02_538906639_m
pyramidal	neocortex	human	Allen Cell Types	H16-06-004-01-13-03_539990522_m
pyramidal	neocortex	human	Allen Cell Types	H16-06-004-02-13-04_538906871_m
pyramidal	neocortex	human	Allen Cell Types	H17-03-002-11-04-02_596792557_m
pyramidal	neocortex	human	Allen Cell Types	H17-03-002-11-04-04_603514410_m
pyramidal	neocortex	human	Allen Cell Types	H17-03-002-11-06-05_603514429_m
pyramidal	neocortex	human	Allen Cell Types	H17-06-003-11-05-01_605485079_m
pyramidal	neocortex	human	Allen Cell Types	H17-06-004-11-05-04_599474134_m
pyramidal	neocortex	human	Allen Cell Types	H17-06-004-11-05-05_605857665_m
pyramidal	neocortex	human	Allen Cell Types	H17-06-004-11-05-06_605851449_m
pyramidal	neocortex	human	Allen Cell Types	H17-06-005-12-10-07_605485535_m
pyramidal	neocortex	human	Allen Cell Types	H17-06-005-12-16-03_606347962_m
pyramidal	neocortex	human	Allen Cell Types	H17-06-006-11-08-04_585893370_m
pyramidal	neocortex	human	Allen Cell Types	H17-06-006-11-08-08_601946464_m
pyramidal	neocortex	human	Allen Cell Types	H17-06-006-11-09-04_591274508_m
pyramidal	neocortex	human	Allen Cell Types	H17-06-009-11-04-04_614429153_m

Table 9. Data Sources for Pyramidal Tumor Cells - Machine Learning Classification

Cell Type	Region	Species	Archive Name	File Name
pyramidal	neocortex	human	Allen Cell Types	576110753_transformed
pyramidal	neocortex	human	Allen Cell Types	576118161_transformed
pyramidal	neocortex	human	Allen Cell Types	576134298_transformed
pyramidal	neocortex	human	Allen Cell Types	576140393_transformed
pyramidal	neocortex	human	Allen Cell Types	665713811_transformed
pyramidal	neocortex	human	Allen Cell Types	716918890_transformed
pyramidal	neocortex	human	Allen Cell Types	716929071_transformed
pyramidal	neocortex	human	Allen Cell Types	768819569_transformed
pyramidal	neocortex	human	Allen Cell Types	768848167_transformed
pyramidal	neocortex	human	Allen Cell Types	768867010_transformed
pyramidal	neocortex	human	Allen Cell Types	768885440_transformed
pyramidal	neocortex	human	Allen Cell Types	768904007_transformed
pyramidal	neocortex	human	Allen Cell Types	769228370_transformed

Table 10. Data Sources for Pyramidal Epilepsy Cells - Machine Learning Classification

Cell Type	Region	Species	Archive Name	File Name
pyramidal	neocortex	human	Allen Cell Types	541557114_transformed
pyramidal	neocortex	human	Allen Cell Types	571732727_transformed
pyramidal	neocortex	human	Allen Cell Types	592532014_transformed
pyramidal	neocortex	human	Allen Cell Types	595572609_transformed
pyramidal	neocortex	human	Allen Cell Types	596898838_transformed
pyramidal	neocortex	human	Allen Cell Types	677088033_transformed
pyramidal	neocortex	human	Allen Cell Types	689306818_transformed
pyramidal	neocortex	human	Allen Cell Types	695521538_transformed
pyramidal	neocortex	human	Allen Cell Types	720828444_transformed
pyramidal	neocortex	human	Allen Cell Types	720862326_transformed
pyramidal	neocortex	human	Allen Cell Types	737089555_transformed
pyramidal	neocortex	human	Allen Cell Types	737134157_transformed
pyramidal	neocortex	human	Allen Cell Types	767433014_transformed
pyramidal	neocortex	human	Allen Cell Types	767829778_transformed
pyramidal	neocortex	human	Allen Cell Types	770255008_transformed
pyramidal	neocortex	human	Allen Cell Types	774420848_transformed
pyramidal	neocortex	human	Allen Cell Types	774620186_transformed
pyramidal	neocortex	human	Allen Cell Types	787239157_transformed
pyramidal	neocortex	human	Allen Cell Types	794276683_transformed
pyramidal	neocortex	human	Allen Cell Types	832627767_transformed
