# Supplementary material for: Information flow drives localized morphological differences across neuronal and glial cell types
Source: Front Comput Neurosci. 2026 Mar 11;20:1771227. doi: 10.3389/fncom.2026.1771227 (PMC13013023; doi:10.3389/fncom.2026.1771227)
Supplement: Supplementary file 2 [file Data_Sheet_2.pdf]

Supplementary Material

Paheli Desai-Chowdhry [1,5*], **Alexander B Brummer** [3], **Samhita Mallavarapu** [1,4], **Masai Oakes** [5], **and Van Savage** [1,2,6]

[1] *University of California Los Angeles, Department of Computational Medicine, Los Angeles, California, United States of America*
[2] *University of California Los Angeles, Department of Ecology and Evolutionary Biology, Los Angeles, California, United States of America*
[3] *College of Charleston, Department of Physics and Astronomy, Charleston, South Carolina, United States of America*
[4] *Tufts University, Department of Computer Science, Medford, Massachusets, United States of America*
[5] *Trinity Washington University, Department of Mathematics, Washington, District of Columbia, United States of America*
[6] *Santa Fe Institute, Santa Fe, New Mexico, United States of America*

Correspondence*:
Corresponding Author
desai-chowdhryp@trinitydc.edu

Here, we show the extended analysis for dendrite cell-type classification as well as applying these methods to analyze differences between healthy cells and cells from tumor and epilepsy patients. For the comparison of diseased cells to healthy cells in particular, there are differences that occur at specific leaf numbers, meaning different locations in the cell. Our results thus suggest that the morphological distinctions between cells are driven by information flow at localized cell regions for these cells. While our preliminary results for the latter are promising, further studies are needed to validate the conclusions.

1 EXTENDED DENDRITE COMPARISONS

Here, we compare 4 different types of dendrites—Motoneurons, Purkinje cells, Medium Spiny Neurons, and Pyramidal cells—for a total of 6 comparisons. We first compare these dendrites using $\bar{\beta}$ and $\Delta\beta$ as features, as shown in Figure 1. The performance of these methods are measured by the AUC (area under curve) of the ROC curves, which are also shown in Figure 2. Next, we compare these dendrites using $\bar{\beta}$, $\Delta\beta$, and $L_{n,rel}$ as features, as shown in Figure 3. The ROC curves for this feature space are also shown in 4. The data in Tables 1-6 of the main text are based on the combined branching point data for all images.

For all of these comparisons, the 7 classification methods perform relatively well, and incorporating $L_{n,rel}$ as an additional feature in the classification improves the performance, though the improvement is most significant for the comparison of Medium Spiny Neurons and all the other three cells. The classification method that consistently performed the best for all comparisons was Random Forest.

We can summarize the results using Figure **??**, which shows the strength of the classification performance, where the line thickness between each pair is proportional to the AUC.

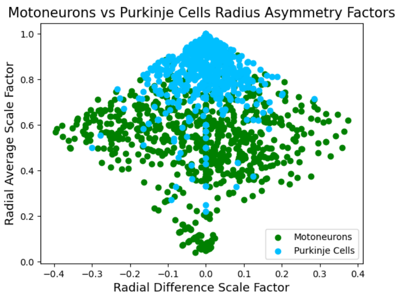

Figure 1a. Motoneurons versus S Purkinje Cells

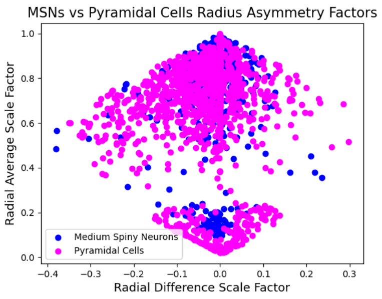

Figure 1b. MSNs versus Pyramidal Cells

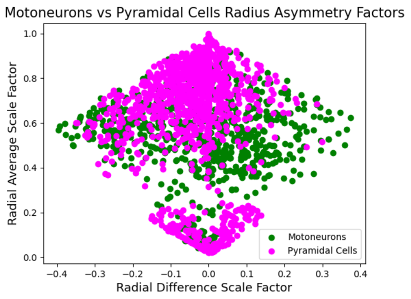

Figure 1c. Motoneurons versus Pyramidal Cells

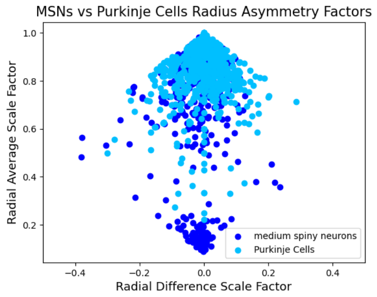

Figure 1d. MSNs versus Purkinje Cells

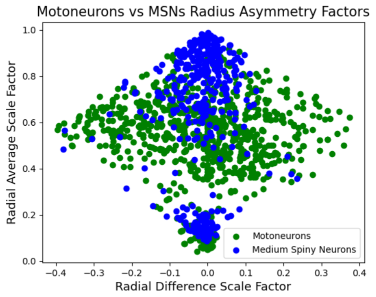

Figure 1e. Motoneurons versus MSNs

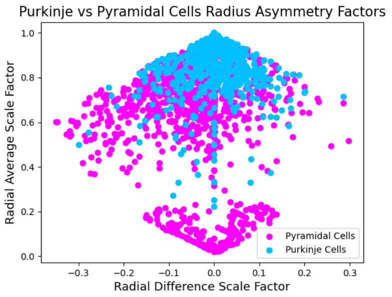

Figure 1f. Purkinje versus Pyramidal Cells

Figure 1. Plots of 2-dimensional feature spaces of the training data for 6 different combinations of dendritic types. In each of these plots, one can observe clear distinctions between the cell types, evidenced by the separate clusters of data points of each of the assigned colors.

Table 1. Healthy versus Diseased Neurons Classification AUC (Area Under ROC curve) Measures The AUC (area under the curve) values corresponding to the ROC curves show the strongest performance when they are closest to 1. The filtered data focused on the clusters of data that are localized near the soma, at $L_{n,rel} = 0$. Here, we can see that filtering the data significantly increases the AUC for all methods. However, for the filtered data, there is more uncertainty in the AUC measures due to the more limited number of data points.

Type/Method	LR	SVM	KNN	RF	DT	Bayes	NN
Tumor	0.584 ± 0.072	0.662 ± 0.070	0.681 ± 0.067	0.598 ± 0.075	0.566 ± 0.073	0.645 ± 0.069	0.523 ± 0.072
Tumor, Filtered	0.692 ± 0.312	0.731 ± 0.268	0.529 ± 0.291	0.740 ± 0.232	0.726 ± 0.222	0.778 ± 0.216	0.558 ± 0.314
Epilepsy	0.563 ± 0.057	0.661 ± 0.054	0.608 ± 0.056	0.748 ± 0.047	0.699 ± 0.051	0.588 ± 0.056	0.585 ± 0.049
Epilepsy, Filtered	0.668 ± 0.186	0.806 ± 0.145	0.827 ± 0.136	0.812 ± 0.132	0.868 ± 0.117	0.796 ± 0.171	0.796 ± 0.158

2 HEALTHY VERSUS DISEASED CELLS

Here, we apply these methods to attempt to distinguish between healthy and diseased dendrites in humans. We compare control cells for Pyramidal principle cells in the Middle Temporal Gyrus to two different classes of diseased cells—cells from patients with tumors and cells from patients with epilepsy. Table 1 shows the performance of 7 different classification methods, all using $\bar{\beta}$, $\Delta\beta$, and $L_{n,rel}$ as features. The performance of these classification methods are measured by the AUC (area under curve) of the ROC curves. The data in Table 1 are based on the combined branching point data for all images. Classification

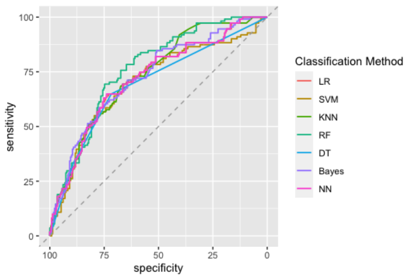

Figure 2a. Motoneurons versus Purkinje Cells

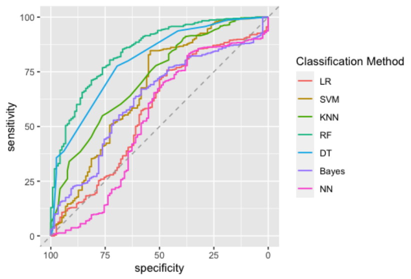

Figure 2b. MSNs versus Pyramidal Cells

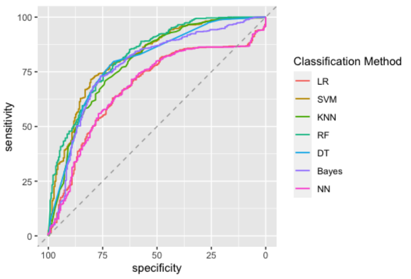

Figure 2c. Motoneurons versus Pyramidal Cells

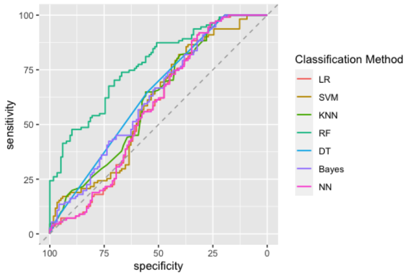

Figure 2d. MSNs versus Purkinje Cells

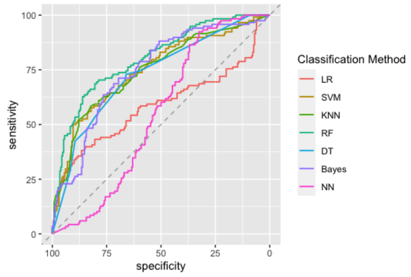

Figure 2e. Motoneurons versus MSNs

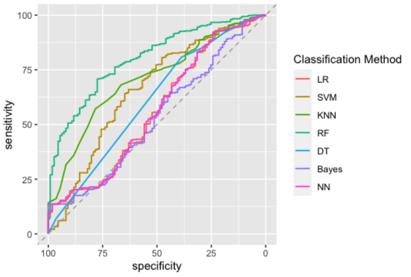

Figure 2f. Purkinje versus Pyramidal Cells

Figure 2. Plots of ROC curves illustrating the performance of classification methods on the test data for 6 different combinations of dendrite cell-type comparisons, using $\overline{\beta}$ and $\Delta\beta$ as features. ROC curves show the tradeoff between true positive rates and false positive rates, which is a more accurate assessment of the performance of classifications than accuracy especially for datasets that are not balanced. The AUC (area under the curve) of these ROC curves show the strongest performance when they are closest to 1, and the curves that are closer to the top left corner at the intersection of axes than the horizontal dashed line illustrate better performance. We can observe the green ROC curves, corresponding to the Random Forest (RF) method, consistently on top of the set of curves, showing its superior performance in classification.

of the cell-types in the test sets based on the whole images, or the average prediction of each of the points in the images, is shown in Table 2.

Table 2. Image Based Classification Accuracy Measures This table shows the accuracy measures of classification using combined data from all the individual data points to classify each image or whole neuron based on average classification of all branching points in the image for each method. Out of all the images with branching junction data that fit our precision requirements for the pixel sizes, we balanced the sets of images and randomly assigned 70-80 % of the images to training data and the remaining images to test data. The numbers of images here for each classification (comparison between two groups) reflects the number of images in those test sets.

Classification/Method	LR	SVM	KNN	**RF**	DT	Bayes	NN
Tumor/Control	3/6 images	3/6 images	3/6 images	4/6 images	3/6 images	3/6 images	4/6 images
Tumor-Filtered/Control	3/6 images	5/6 images	2/6 images	4/6 images	5/6 images	4/6 images	4/6 images
Epilepsy/Control	5/10 images	5/10 images	6/10 images	9/10 images	6/10 images	6/10 images	7/10 images
Epilepsy-Filtered/Control	6/10 images	6/10 images	7/10 images	7/10 images	7/10 images	5/10 images	8/10 images

We show both the 2-dimensional and 3-dimensional feature spaces for both these comparisons in Figure 6. As seen in these images, we can observe a significant distinction between the control and diseased cells

in the data for both types, visible as clusters of the data with both low $\bar{\beta}$ and low $L_{n,rel}$ values. We filter the data to focus on the points with $L_{n,rel} = 0$, or the first branching junction in the tree from the soma, and then perform the classification methods again. This approach leads to better performance, although there is more uncertainty in the AUC values due to the limited number of data points. The performance of the classification methods using the filtered data is also reported in Table 1. We notice in the data that in this cluster where the distinction is observed, the $\Delta\beta$ values for the controls are closer to 0, or symmetric branching junctions, whereas the data for the diseased cells are significantly more asymmetric.

Due to the promise of these methods in classifying different types of neuronal and glial cell types, we apply these methods to look at distinctions between healthy cells and diseased cells in order to attempt to extract insights about the pathology. In this study, we focus on comparisons between control cells and cells from patients with tumors and epilepsy. Interestingly, although the classification methods were minimally successful in separating the diseased branching junctions from healthy branching junctions in the raw data, we observe that there is a clear distinction in the data localized at the soma. If we filter the data based on $L_{n,rel}$, focusing only on the data points with $L_{n,rel} = 0$, the classification methods perform much better. Observing the data, we notice that for the branching junctions at the soma, for the control cells, the $\Delta\beta$ values are centered closer to 0 (the symmetric case), whereas for both the tumor and epilepsy data, the $\Delta\beta$ values diverge from 0 (suggesting asymmetry). We illustrate this observed phenomenon in Figure 7. This difference between healthy and diseased cells is localized near the soma, and suggests that there might be a potential biomarker to identify these diseased cells that is localized near the soma.

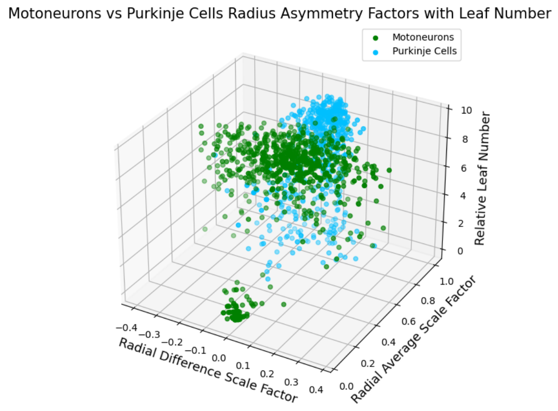

Figure 3a. Motoneurons versus Purkinje Cells

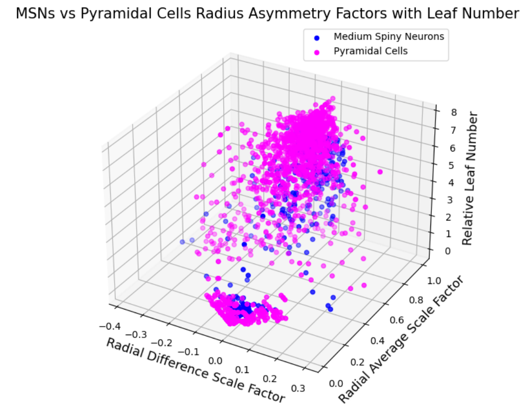

Figure 3b. MSNs versus Pyramidal Cells

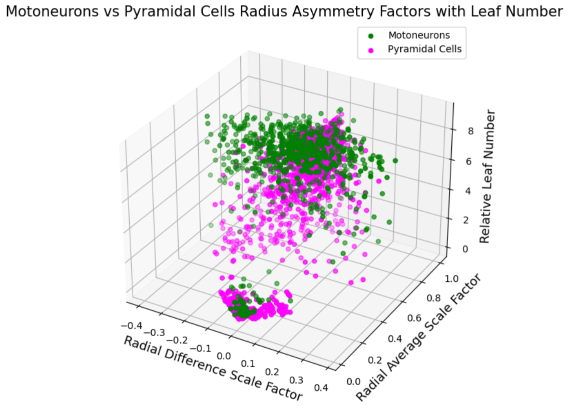

Figure 3c. Motoneurons versus Pyramidal Cells

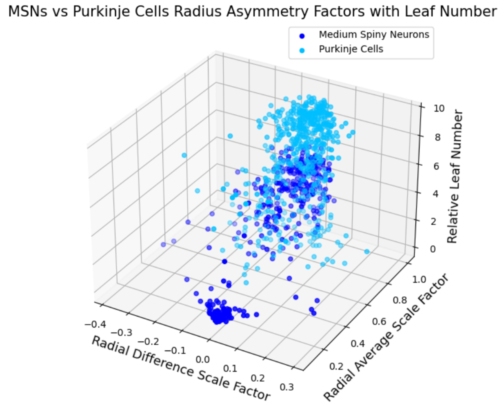

Figure 3d. MSNs versus Purkinje Cells

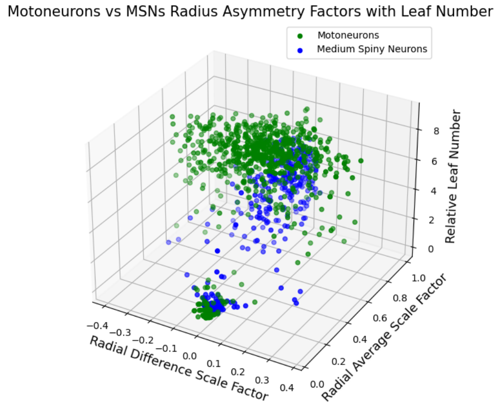

Figure 3e. Motoneurons versus MSNs

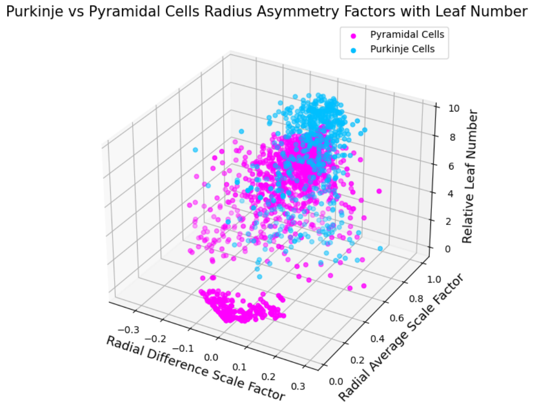

Figure 3f. Purkinje versus Pyramidal Cells

Figure 3. Plots of 3-dimensional feature spaces of the training data for 6 different combinations of dendritic types. In each of these plots, one can observe clear distinctions between the cell types, evidenced by the separate clusters of data points of each of the assigned colors. These clusters show even greater distinctions than those observed for the 2-dimensional feature spaces.

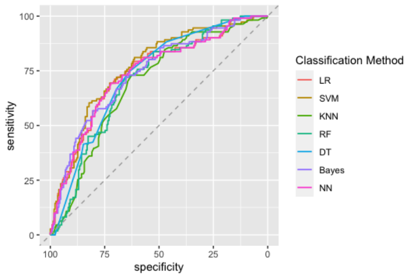

Figure 4a. Motoneurons versus Purkinje Cells

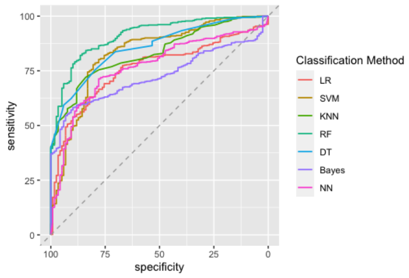

Figure 4b. MSNs versus Pyramidal Cells

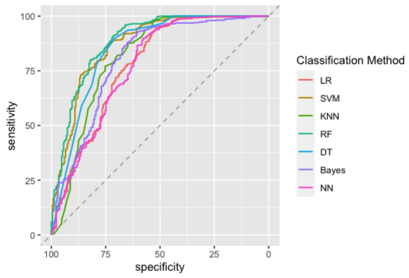

Figure 4c. Motoneurons versus Pyramidal Cells

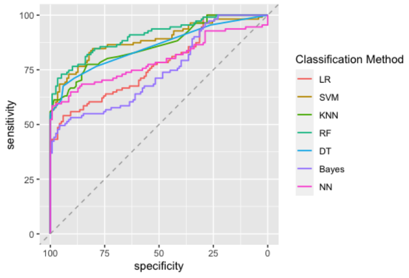

Figure 4d. MSNs versus Purkinje Cells

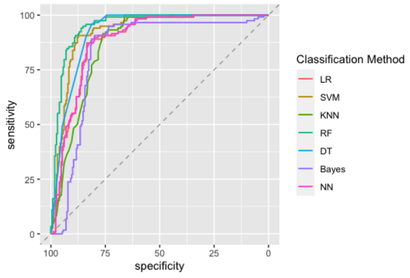

Figure 4e. Motoneurons versus MSNs

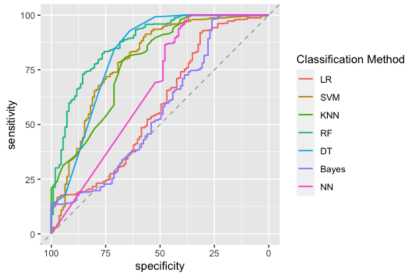

Figure 4f. Purkinje versus Pyramidal Cells

Figure 4. Plots of ROC curves illustrating the performance of classification methods on the test data for 6 different combinations of dendrite cell-type comparisons, using $\overline{\beta}$, $\Delta\beta$, and $L_{n,rel}$ as features. ROC curves show the tradeoff between true positive rates and false positive rates, which is a more accurate assessment of the performance of classifications than accuracy especially for datasets that are not balanced. The AUC (area under the curve) of these ROC curves show the strongest performance when they are closest to 1, and the curves that are closer to the top left corner at the intersection of axes than the horizontal dashed line illustrate better performance. We can observe the green ROC curves, corresponding to the Random Forest (RF) method, consistently on top of the set of curves, showing its superior performance in classification. Moreover, we can observe that these ROC curves tend to show better performance than those observed previously that did not include $L_{n,rel}$ as a feature.

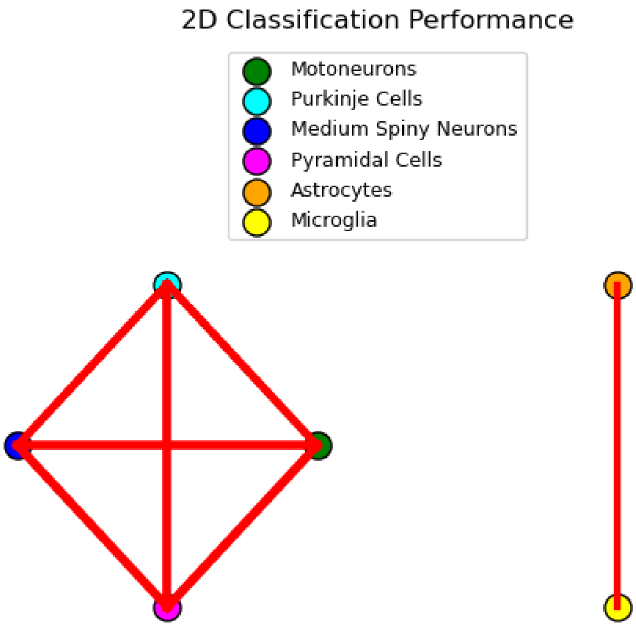

Figure 5a. 2D Results

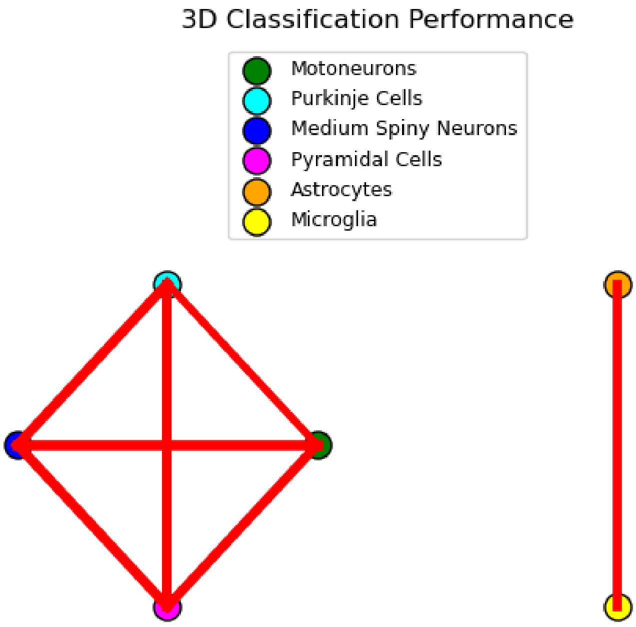

Figure 5b. 3D Results

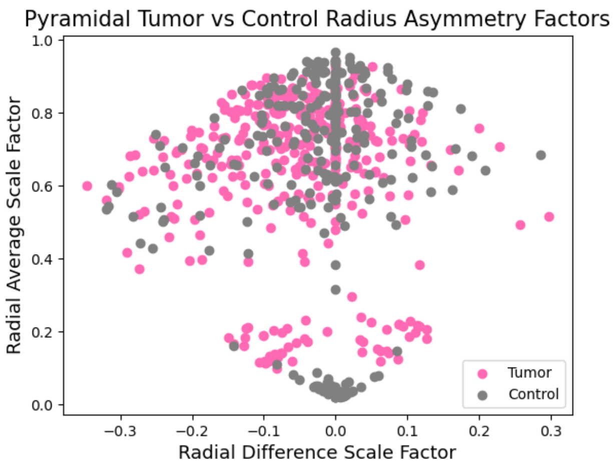

Figure 6a. 2-dimensional feature space: Tumor

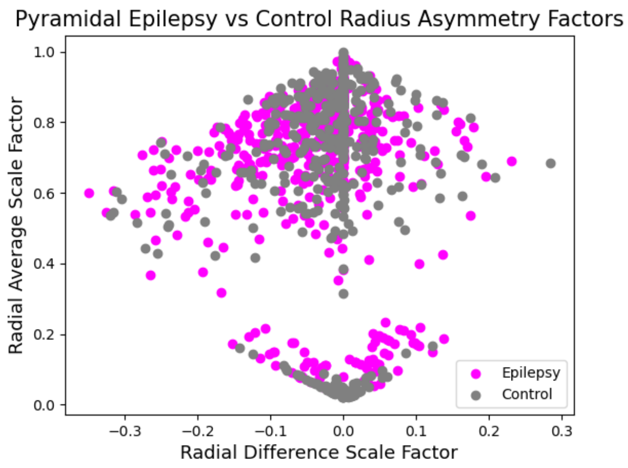

Figure 6b. 2-dimensional feature space: Epilepsy

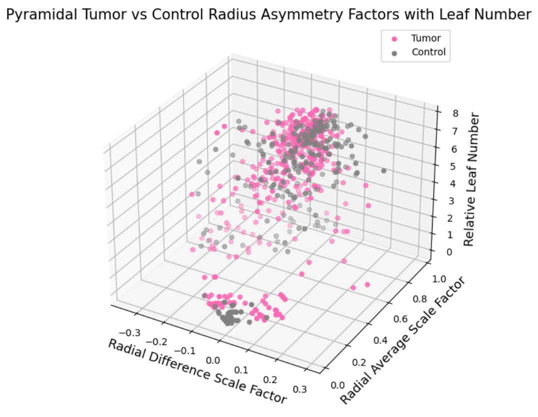

Figure 6c. 3-dimensional feature space: Tumor

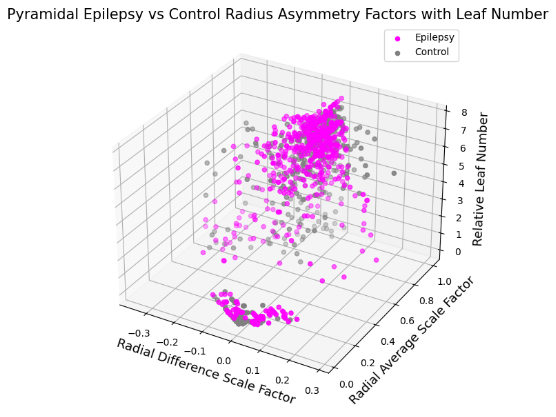

Figure 6d. 3-dimensional feature space: Epilepsy

Figure 6. Plots of feature spaces showing the comparison of training data for control and diseased cells, tumor and epilepsy cells, with **(A)**, **(B)** $\overline{\beta}$ and $\Delta\beta$, and **(C)**, **(D)** $\overline{\beta}$, $\Delta\beta$, and $L_{n,rel}$ as features. One can observe clear distinctions between the tumor and epilepsy cells and the control cells, evidenced by the separate clusters of data points of each of the assigned colors. For both the 2-dimensional and 3-dimensional feature spaces, we observe that the distinctions between the types are localized to regions with both low $\overline{\beta}$ and low $L_{n,rel}$ values. Thus, we filter the data to focus on these clusters to classify between cell types.

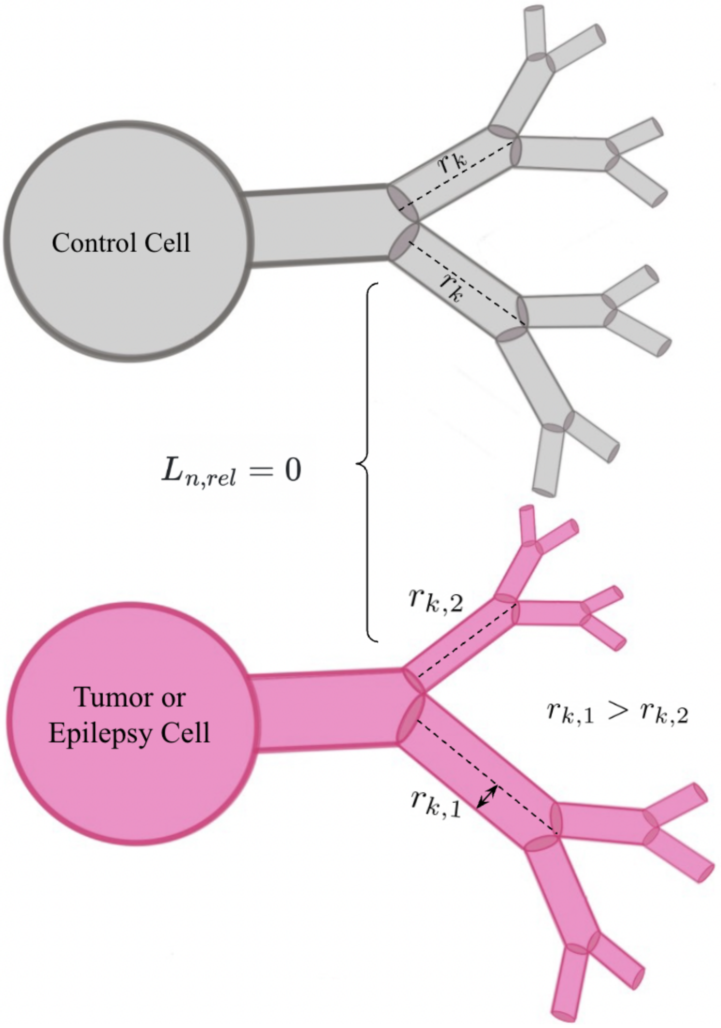

Figure 7. Visualization of the observed differences between control and tumor/epilepsy human pyramidal cells. Since the $\Delta\beta$ values at $L_{n,rel}$ values of 0 for the controls are closer to zero, more symmetric branching junctions occurs near the soma. Meanwhile, the data for the diseased cells shows $\Delta\beta$ values that vary from 0 at $L_{n,rel}$ values of 0, suggesting that more asymmteric branching junctions occur closer to the soma.
